# A Rational Design of Metal–Organic Framework Nanozyme with High-Performance Copper Active Centers for Alleviating Chemical Corneal Burns

**DOI:** 10.1007/s40820-023-01059-9

**Published:** 2023-04-30

**Authors:** Yonghua Tang, Yi Han, Jiachen Zhao, Yufei Lv, Chaoyu Fan, Lan Zheng, Zhisen Zhang, Zuguo Liu, Cheng Li, Youhui Lin

**Affiliations:** 1 Department of Physics, Research Institute for Biomimetics and Soft Matter, Fujian Provincial Key Laboratory for Soft Functional Materials Research, Xiamen University, Xiamen, 361005 People’s Republic of China; 2 Fujian Provincial Key Laboratory of Ophthalmology and Visual Science & Ocular Surface and Corneal Diseases, Eye Institute & Affiliated Xiamen Eye Center, School of Medicine, Xiamen University, Xiamen, 361102 People’s Republic of China; 3 National Institute for Data Science in Health and Medicine, Xiamen University, Xiamen, 361102 People’s Republic of China; 4 Postdoctoral Mobile Station of Basic Medical Sciences, Hengyang Medical School, Department of Ophthalmology, The First Affiliated Hospital of University of South China, University of South China, Hengyang, Hunan 421001 People’s Republic of China

**Keywords:** Metal–organic frameworks nanozyme, Superoxide dismutase, Halogen, Chemical ocular burn, Corneal diseases

## Abstract

Metal–organic frameworks (MOFs) have attracted significant research interest in biomimetic catalysis. However, the modulation of the activity of MOFs by precisely tuning the coordination of metal nodes is still a significant challenge. Inspired by metalloenzymes with well-defined coordination structures, a series of MOFs containing halogen-coordinated copper nodes (Cu-X MOFs, X = Cl, Br, I) are employed to elucidate their structure–activity relationship. Intriguingly, experimental and theoretical results strongly support that precisely tuning the coordination of halogen atoms directly regulates the enzyme-like activities of Cu-X MOFs by influencing the spatial configuration and electronic structure of the Cu active center. The optimal Cu–Cl MOF exhibits excellent superoxide dismutase-like activity with a specific activity one order of magnitude higher than the reported Cu-based nanozymes. More importantly, by performing enzyme-mimicking catalysis, the Cu–Cl MOF nanozyme can significantly scavenge reactive oxygen species and alleviate oxidative stress, thus effectively relieving ocular chemical burns. Mechanistically, the antioxidant and antiapoptotic properties of Cu–Cl MOF are achieved by regulating the NRF2 and JNK or P38 MAPK pathways. Our work provides a novel way to refine MOF nanozymes by directly engineering the coordination microenvironment and, more significantly, demonstrating their potential therapeutic effect in ophthalmic disease.

**Supplementary Information:**

The online version contains supplementary material available at 10.1007/s40820-023-01059-9.

## Highlights

- Inspired by metalloenzymes with well-defined coordination structures, a series of Cu-X metal–organic frameworks (MOFs) nanozymes with tunable copper active centers were successfully constructed.
- Experimental and theoretical results strongly supported that precisely tuning the coordination of halogen atoms could directly regulate the enzyme-like activities of Cu-X MOFs by influencing their spatial configuration and electronic structure.
- The optimal Cu–Cl MOF with excellent enzyme-mimicking activities could effectively relieve ocular chemical burns by possible antioxidant and antiapoptotic mechanisms.

**Supplementary Information:**

The online version contains supplementary material available at 10.1007/s40820-023-01059-9.

## Graphical abstract

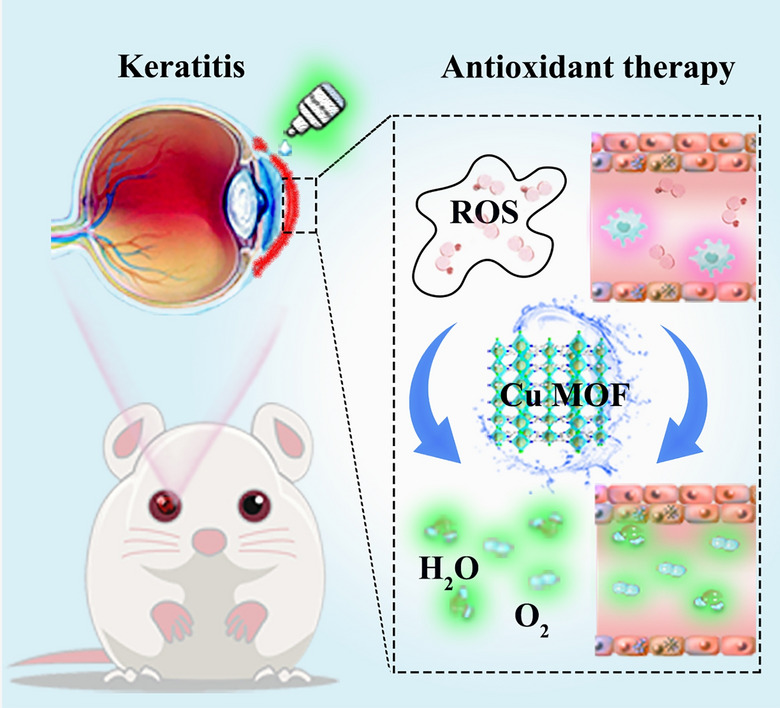

## Introduction

Chemical ocular burns occur in 10–22% of all ocular traumas, causing more than 20 million dollars in emergency room visits yearly and generating widespread public health concerns [1]. One of the main features of chemical burns is the promotion of high levels of reactive oxygen species (ROS) that lead to the development of oxidative stress on the ocular surface, which exceeds the antioxidant capacity and eventually induces cell death and inflammation [2]. Traditional strategies based on citrate, antibiotics, hyaluronic acid, dimethyl thiourea, allopurinol, and acetylcysteine in the treatment of oxidative disorders after corneal alkali burns are limited owing to their high cost, high toxicity, and low efficiency [3]. Therefore, developing new drugs or materials with high antioxidant efficiency and good biosafety remains an important research direction.

Artificial enzyme catalysis has been an exciting research subject due to its great potential to provide unparalleled high reactivity and selectivity for many essential reactions and significantly reduce the cost of using natural enzymes [4–8]. As the most prominent representatives of artificial enzymes, nanozymes have shown a wide range of applications from in vitro assays to disease treatment by combining their unique physicochemical properties and catalytic activity [9–16]. MOFs are commonly used to construct nanozymes that mimic chemical processes, such as redox reactions catalyzed by biological redox enzymes [17–19]. However, most reported MOFs usually possess relatively low catalytic activity compared with metalloenzymes. Therefore, significant effort has been focused on developing the MOFs with high performance. Among the many available approaches, tuning the linker substituents in MOFs has attracted considerable interest since it provides a critical opportunity to alter the electronic structure of nodal metals and thereby modulate their mimetic enzyme activity [20, 21]. Although promising, precise control of the positions of these substituents remains a considerable challenge. Additionally, such indirect control is challenging due to a significant change in the electrical structure of the nodal metal [21, 22]. Therefore, it is urgent to find a very effective and direct modulation strategy to enhance the activity of MOFs.

Recently, experimental and computational studies have shown that surface-modified halogen atoms can improve the reactivity of the active sites of heterogeneous catalysts, such as metal, metal oxide, and carbon-based catalysts [23, 24]. However, the surface modification strategy by halogen atoms can lead to instability and a limited effect on their activity. Furthermore, there is a possibility that the halogen atoms can serve as a structural component in the active site of catalysts, which can remarkably modulate the catalyst’s activity. To our knowledge, in MOFs with halogen-coordinated metal nodes, halogens not only participate in forming crystals as essential components but can also directly modulate the metal center’s electronic structure. However, the physicochemical properties of such MOFs remain largely unexplored, and there is little understanding of the importance of halogens on their reactivity at the atomic level.

**Fig. 1:**
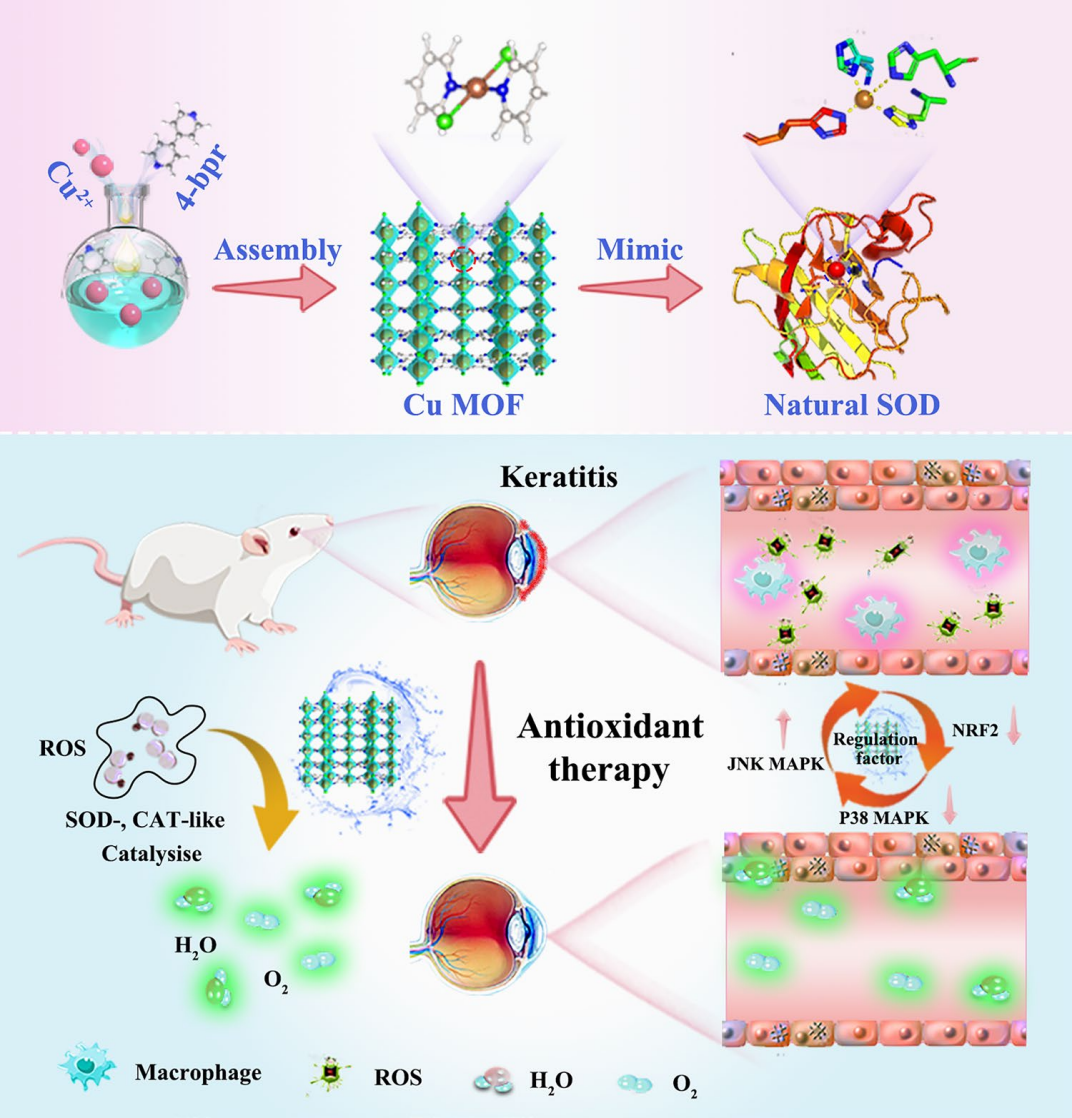
Schematic diagram of the synthesis of Cu MOF nanozymes mimicking superoxide dismutase for the therapy of chemical corneal burns

In the present work, we proposed a straightforward and robust procedure to fabricate excellent copper active centers in MOFs to alleviate chemical corneal burns. Specifically, a series of MOFs with different halogen-coordinated copper nodes (Cu-X MOFs, X = Cl, Br, I) were constructed by the facile self-assembly of Cu ions, halogen ions, and 4,4’-bipyridine. Consequently, by precisely tuning the coordination of halogen atoms, Cu–Cl MOFs with optimal enzyme-like activities could breakdown ROS into H_2_O and O_2_ (Fig. 1). Density functional theory (DFT) calculations were also employed to reveal the origin of the superoxide dismutase (SOD)-like activity of Cu-X MOFs and the Regulatory mechanism of their activity by halogens. More importantly, due to their intrinsic enzyme-like activities to scavenge ROS, Cu–Cl MOFs exhibited significant antioxidant and antiapoptotic functions in human corneal epithelial (HCE) cells by regulating NRF2 and JNK or P38 MAPK in vitro. Moreover, as proof of concept, pathological corneal damage treated with Cu–Cl MOFs was effectively alleviated in an animal model of chemical corneal burn. In addition, Cu-Cl MOFs in the form of eye drops are safe for the ocular surface. This study proposes a new idea to optimize the catalytic performance of MOF-based nanozymes and establishes a structure–activity relationship that directly regulates the catalytic effect of the metal active centers. Furthermore, highly active Cu–Cl MOF nanozymes can present a potential therapeutic option for treating severe corneal disease.

## Experimental and Methods

### Materials

All chemicals were of analytical grade and were used without further treatment. Phosphate buffer solution (PBS, 0.1 M) was prepared by mixing NaH_2_PO_4_ and Na_2_HPO_4_, obtained from Macklin. CuSO_4_·5H_2_O (99%), NaCl, NaBr, NaI, 4,4’-bipyridine, and ethanol were bought from Macklin. Hydrogen peroxide (H_2_O_2_, 30 wt%) was purchased from Beijing Chemicals (Beijing, China) and stored in a 4 °C refrigerator. Ultrapure water was used to prepare all aqueous solutions throughout a Millipore system (18.2  MΩ; Millipore Co., USA).

### Characterization

A PANalytical X-ray diffractometer recorded the phase structures of the resultant catalysts with monochromatized Cu Kα radiation (λ = 1.5406 Å), and room temperature UV − vis absorption spectroscopy (DRS) of the samples was conducted on a Lambda 750, PerkinElmer. Samples were subjected to annular dark-field imaging in a JEM1400 plus transmission electron microscope operated at an acceleration voltage of 100 kV. These nanosheet samples were first dispersed in EtOH and then deposited onto a carbon-coated copper grid for high-resolution TEM (JEM-2100F) imaging operating at 200 kV and EDX mapping. X-ray photoelectron spectroscopy (XPS) studies were performed on an Escalab 250Xi (Thermo Fisher Scientific). Thermogravimetric analysis was conducted on a NETZSCH STA 449F3 instrument, the temperature was raised from room temperature to 700 °C, and the heating rate was 10 K min^−1^ in nitrogen.

### Density Functional Theory Calculations

First-principles calculations were performed within the density generalized function theory framework using the Vienna Ab initio Simulation Package (VASP) [25, 26]. The Perdew–Burke–Enzerhof (PBE) [27] generalized and projection-enhanced wave (PAW) methods [28] describe the exchange–correlation potential and ion–electron interactions. The adopted DFT-D3 method considered van der Waals (vdW) interactions [29]. The cutoff energy of the plane wave base was chosen to be 500 eV, and the Brillouin zone was sampled using a 2 × 2 × 7 Monkhorst–Pack k-point grid for 1 × 1 Cu-X MOFs supercell (CCDC: No.1271445) and 2 × 2 × 1 for 1 × 1 slab (001). The energy and force convergence criteria were 10^–5^ eV and 0.03 eV Å^−1^, respectively. The vacuum layer was set to 15 Å in the z-direction to avoid interactions between the adjacent single-molecule layers. Molecular species, such as H_2_O, H_2_O_2_, and •OOH, were simulated in a 15 Å cubic lattice using the same cutoff parameters and convergence criterion as the slab.

The adsorption energies were calculated using Eq. 1:

1 $$E_{{{\text{ads}}}} = E_{{\text{slab + mol }}} - E_{{{\text{slab}}}} - E_{{{\text{mol}}}}$$

where, *E*_slab+mol_ denoted the energy of the Cu-X MOFs slab with the molecular adsorbate, and *E*_slab_ and *E*_mol_ are the energies of the isolated slab and adsorbate, respectively. The charge density difference was evaluated by Eq. 2:

2 $$\Delta_{\rho } = \rho_{A + B} - \rho_{A} - \rho_{B}$$

where, *ρ*_X_ is the electronic charge density of *X* (*X* = *A*, *B*, *A* + *B*). The *E*_vs HE_ was evaluated by Eq. 3:

3 $$E_{{{\text{vs}}\;{\text{ HE}}}} = E_{{{\text{vac}}}} - E_{{{\text{vs }}\;{\text{fermi}}}} - E_{{{\text{fermi}}}} - 4.5$$

where, *E*_vs fermi_ is the energy band (*E*_vs fermi_) of the material relative to the Fermi energy (*E*_fermi_), and 4.5 eV is the scaling factor related to the normal hydrogen electrode scale (NHE) and the absolute vacuum scale (AVS).

### Preparation of the Cu-X MOFs Nanozymes

Cu-X MOFs were self-assembled at room temperature using CuSO_4_·5H_2_O, NaX (*X* = Cl, Br, I), and 4,4’-bipyridine [30]. Typically, 1 mL of 4,4’ -bipyridine (100 mM) was dissolved in anhydrous ethanol and added dropwise to 9 mL of aqueous Cu^2+^ (5.6 mM) and X^−^ (11.2 mM) and stirred (400 r min^−1^) well. After stirring (400 r min^−1^) the reaction at room temperature for 1 h, the precipitate was collected by centrifugation and washed three times with ultrapure water to remove the unreacted reagents.

### SOD-Like Activity of Cu-X MOFs Nanozymes

The SOD-like activity of Cu-X MOFs was assessed by monitoring the amount of •O_2_^−^ scavenged by INT probe. Initially, xanthine (0.6 × 10^–3^ M) and xanthine oxidase (0.05 U mL^−1^) were mixed in 1 mL of phosphate buffer (0.1 M, pH 7.4) to generate •O_2_^−^for 5 min. Subsequently, different volumes of Cu-X MOFs (1 mg mL^−1^: 5, 10, 20, 50, 100, and 200 µL) were added and undisturbed for 5 min. Finally, the remaining •O_2_^−^ was detected by INT probe. The •O_2_^−^ reduced INT to a red product with an absorption peak at 500 nm, which was determined by UV–vis absorption spectroscopy. The proportion of the •O_2_^−^ was calculated by Eq. 4:

4 $${\text{Elimination}}\left( {\text{\% }} \right) = \left[ {\frac{{A_{1} - A_{2} }}{{A_{1} - A_{0} }}} \right] \times 100{\text{\% }}$$

where, *A*_0_ is the absorbance of INT, and *A*_1_ and *A*_2_ represent the absorbance at 500 nm without Cu-X MOFs and in the presence of Cu-X MOFs, respectively.

### Catalase-Like Activity of Cu-X MOFs Nanozymes

The catalase (CAT)-like activity of Cu-X MOFs nanozymes was measured at room temperature using a dissolved oxygen meter (JPSJ-605, Leici. China). Typically, experiments were performed using 20 µg mL^–1^ of Cu-X MOFs nanozymes mixed with different concentrations of H_2_O_2_ in 10.0 mL of phosphate buffer (0.1 M, pH 7.4). Cu-X MOFs nanozymes catalyzed the decomposition of H_2_O_2_ to H_2_O and O_2_, which proved the CAT-like activity of Cu-X MOFs nanozymes.

### Hydroxyl Radical Scavenging Activity of Cu-X MOFs Nanozymes

The •OH radical was generated by the Fenton reaction of 0.3 × 10^–3^ mM H_2_O_2_ and 0.5 × 10^–3^ mM FeSO_4_ for 1.5 min. Then, 1 mL of Cu-X MOFs nanozymes (166.7 µg mL^−1^) was added to the solution and incubated for another 1.5 min to eliminate •OH. Finally, 0.3 × 10^–4^ M salicylic acid (SA) was introduced to detect residual •OH. SA was oxidized by •OH to 2, 3-dihydroxybenzoic acid with a purple colour product having an absorption peak at 510 nm. The residual •OH was quantified by UV–vis absorption spectrometry. The following equation was used to calculate the removal of •OH:

5 $${\text{Elimination}}\left( {\text{\% }} \right) = \left[ {\frac{{A_{1} - A_{2} }}{{A_{1} - A_{0} }}} \right] \times 100{\text{\% }}$$

where, *A*_0_ is the absorbance of SA and *A*_1,_ and *A*_2_ is the absorbance of 2,3-dihydroxybenzoic acid at 510 nm in the absence and presence of Cu-X MOFs nanozymes.

### Experimental Animal

Female C57BL/6 mice (aged 12–16 weeks and weighed 25–50 g) were acquired from the Animal Center of Xiamen University (Xiamen, Fujian, China) and housed in the animal observation room for ocular research. The animal procedures were supervised by the Experimental Animal Ethics Committee of Xiamen University (No. XMULAC20200170). The protocol followed the Association for Research in Vision and Ophthalmology (ARVO) Statement for the Use of Animals in Ophthalmic and Vision Research. All the mice were raised in familiar surroundings with a humidity environment of 50 ± 10% and a temperature of 23 ± 2 °C. Animals were subjected to 12-h light and 12-h dark cycles, noise less than 60 dB, and free access to standard water and diets.

### Cell Lines and Culture

Human corneal epithelial (HCE) cell lines were purchased from American Type Culture Collection (ATCC, Manassas, VA, USA). HCECs were cultured in Dulbecco's Modified Eagle Medium (DMEM)/Ham's F12 (DMEM/F12, Thermo Fisher Scientific, Waltham, MA, USA) supplemented with hEGF (Peprotech, Rocky Hill, NJ, USA), insulin (Thermo Fisher Scientific), 6% FBS (Thermo Fisher Scientific), and 100 U mL^−1^ penicillin–streptomycin solution (Thermo Fisher Scientific) in an incubator at 37 °C and 5% CO_2_. The cells were passaged by 0.25% trypsin containing 0.02% ethylene diamine tetra-acetic acid (EDTA) (Thermo Fisher Scientific) every 3 days.

### Evaluation of Cell Viability in Vitro

Cell counting kit-8 (CCK-8, Dojindo, Kumamoto, Japan) was employed in this study. HCE cells were seeded on a 96-well plate at 1 × 10^4^ cells per well to evaluate the MOF's cytotoxicity. After cells reached 80% confluence, the medium was replaced by serum-free DMEM/F12 with different concentrations of MOF (5, 10, 25, 50, 100 µg mL^−1^) and further incubated for 6, 12, or 24 h. Subsequently, the media was replaced by 10% CCK-8 constituted media, followed by 4 h incubation at 37 °C in the dark. Then the absorbance was measured spectrophotometrically at 450 nm with a Bio Tek ELX800 microplate reader (Bio Tek Instruments, Winooski, VT). In vitro cytoprotective test was performed with MOF and H_2_O_2_. After cells were seeded and reached 80% confluence, the medium was replaced by serum-free DMEM/F12 or serum-free DMEM/F12 containing 200 µM H_2_O_2_ and various concentrations of MOF (5, 10, 25, 50, 75 µg mL^−1^). After cultivation for 24 h, the viability of cells was determined via CCK-8 assays.

### Fluorescent Staining

HCE cells were seeded on 20-mm-diameter coverslips in 12-well plates with 1 × 10^5^ cells per well. As cells reached 80% confluence, the medium was replaced by (1) serum-free DMEM/F12 or serum-free DMEM/F12 containing (2) 75 µg mL^−1^ MOF + 200 µM H_2_O_2_, (3) 50 µg mL^−1^ MOF + 200 µM H_2_O_2_, (4) 25 µg mL^−1^ MOF + 200 µM H_2_O_2_, (5) 10 µg mL^−1^ MOF + 200 µM H_2_O_2_, (6) 5 µg mL^−1^ MOF + 200 µM H_2_O_2_. After incubation for 6 h, the cells were washed with PBS buffer and incubated with serum-free DMEM/F12 containing 10 µM 2,7-dichlorofluorescein diacetate (DCFH-DA; Beyotime Biotechnology, Shanghai, China) for another 0.5 h. Finally, the cells were washed with PBS buffer, fixed in 4% paraformaldehyde, mounted by 4, 6-diamidino-2-phenylindole (DAPI) (H-1200; Vector, Burlingame, CA, USA), and observed on a fluorescence microscope (Leica, DM2500; Leica, Wetzlar, Germany).

### Flow Cytometric Analysis

HCE cells were seeded on 6-well plates at 5 × 10^5^ cells per well after incubation with different formulations for 6 h and stained with DCFH-DA. Cells were filtered by a 70-µm cell strainer (BD Falcon; Becton–Dickinson, Franklin Lakes, NJ, USA). The stained cells were analyzed using CytoFlex S Flow Cytometer (Beckman Coulter, IN, USA). Flow cytometry data were plotted and quantified using FlowJo software version 10 (Tree Star LLC, OR, USA).

### Western Blotting

HCE cells were seeded on 6-well plates at 5 × 10^5^ cells per well. After incubation with different formulations for 6 h, the cells were harvested and dissolved in cold RIPA buffer (Thermo Fisher Scientific) containing a protease inhibitor cocktail and phosphatase inhibitor cocktail (Thermo Fisher Scientific). The protein solutions were extracted, purified, and quantified by a BCA assay kit (Thermo Fisher Scientific). Equal amounts of protein were loaded on the 12.5% tris–glycine gels for separation and were electronically transferred to polyvinylidene fluoride (PVDF) membranes (Millipore, Billerica, MA, USA). After blocking in 5% BSA, the PVDF membranes were incubated with primary antibodies (Table 1) overnight at 4 °C. The following day, the membranes were washed with TBST buffer three times and incubated with secondary antibodies (Table 1) for 1 h. The ChemiDoc XRS System captured the bands (Bio-Rad Laboratories, Inc., Philadelphia, PA, USA). GAPDH (glyceraldehyde-3-phosphate dehydrogenase) served as an internal control. Blot intensity was calculated with ImageJ Software.

**Table 1:** Antibodies for western blotting and immunofluorescent staining

Antibodies	Source	Category	Usage	Host
*Primary antibody*				
NRF2	Proteintech	16,396–1-AP	Western blotting, Immunofluorescent staining	Rabbit
NQO1	Proteintech	11,451–1-AP	Western blotting	Rabbit
Catalase	Proteintech	21,260–1-AP	Western blotting	Rabbit
BCL-2	Abcam	Ab196495	Western blotting	Rabbit
cleaved-caspase 3	CST	9661	Western blotting	Rabbit
p-JNK	ABclonal	AP0631	Western blotting	Rabbit
JNK	ABclonal	A4867	Western blotting	Rabbit
p-P38	CST	4511	Western blotting	Rabbit
P38	CST	8690	Western blotting	Rabbit
EMR1	ABclonal	A1256	Immunofluorescent staining	Rabbit
α-SMA	Abcam	ab7817	Immunofluorescent staining	Mouse
HRP-GAPDH	Proteintech	HRP-60004	Western blotting	Mouse
*Secondary antibody*				
HRP-conjugated anti-rabbit IgG	Sigma-Aldrich	a0545	Western blotting	Goat
Alexa Fluor 488-conjugated anti-mouse IgG	Invitrogen	A-10680	Immunofluorescent staining	Goat
Alexa Fluor 488-conjugated anti-rabbit IgG	Invitrogen	A-21206	Immunofluorescent staining	Donkey

### Terminal Deoxynucleotidyl Transferase-Mediated dUTP Nick-end Labeling (TUNEL) Staining

HCE cells were seeded on 20-mm-diameter coverslips in 12-well plates at 1 × 10^5^ cells per well, followed by incubating with different formulations for 6 h. Subsequently, the cells were fixed, and the TUNEL staining (catalog no. G3250; Promega, Madison, WI, USA) was performed per the manufacturer's instructions. The coverslips were last mounted with DAPI and subjected to fluorescence microscopy. ImageJ Software was used to count the TUNEL-positive cells per field of view, and the percentage of TUNEL-positive cells was calculated by TUNEL-positive cells/DAPI-positive cells (%).

### Immunofluorescence Staining for HCE Cells

HCE cells were seeded on 20-mm-diameter coverslips in 12-well plates with 1 × 10^5^ cells per well, and then incubated with different formulations for 6 h. Subsequently, the cells were fixed in 4% formaldehyde (under 4 °C) for 15 min, incubated in 0.2% Triton X-100 and blocked with 2% bovine serum albumin, followed by incubation at 4 °C with primary antibodies (Table 1) overnight at 4 °C. On the next day, the cells were washed with PBS and incubated with a secondary antibody (Table 1) for one hour at room temperature in the dark, mounted with DAPI solution, and photographed under a laser confocal scanning microscope (Olympus FV1000MPE-B: Olympus).

### Immunofluorescence Staining for Animal Tissue Section

Murine eyeballs were harvested and fixed in 4% paraformaldehyde under 4 °C. Subsequently, the eyeballs were embedded in optimal cutting temperature compound (Tissue-Tek; SAKURA, CA, USA), cut into sagittal Sects. (5-μm thick), placed on glass slides. The slices were fixed and stained as described above (Table 1). Finally, the slices were mounted with DAPI and observed and imaged by a fluorescence microscope (LeicaDM2500).

### Corneal Alkali Burn Model Establishment and Therapeutic Regimen for Eye Drops

Mice were anesthetized using 1% pentobarbital sodium, tropicamide phenylephrine (Santen, Osaka, Japan), and procaine hydrochloride (Alcon, Fort Worth, TX, USA) eye drops were administrated here before the operation. Filter paper discs were prepared using a 2-mm-diameter trephine and sterilized for the next step. 1 µL of a solution of sodium hydroxide at a concentration of 1 mol L^−1^ was dropped onto a filter paper disc. The excess liquid on the mice's ocular surface (or eyelids) was removed before the filter paper discs (with sodium hydroxide solution) were placed on the central cornea for 15 s. The ocular surface was then rinsed with 1 mL PBS 5 times. Mice were kept in a warm blanket until they woke up, and tobramycin eye drops (Alcon) and carbomer eye gel (Bausch & Lomb, Tampa, FL, USA) were applied once. The different concentrations of MOF eye drops (1, 0.5, 0.25 mg mL^−1^) were topically administered (5 μL each eye) on the murine corneal alkali burn model three times daily (09:00 am, 3:00 pm, and 9:00 pm). The model group received the same volume of PBS as a substitute.

### Slit Lamp Examination

To assess corneal transparency and other pathological changes, slit lamp images were noted at different time points. The murine cornea’s symptoms were graded using the criteria in Table 2.

**Table 2:** Grading criteria of the murine corneas

	Grade 0	Grade 1	Grade 2	Grade 3	Grade 4
Area of corneal opacity	0	1–25%	26–50%	51–75%	76–100%
Density of corneal opacity	Not cloudy	Slight cloudiness, pupil visible	Cloudy, mainly pupil discernible	Moderate cloudiness, partial pupil discernible	Cloudy, opacity, pupil invisible

### In Vivo Confocal Microscope Examination

Murine corneas were scanned via an in vivo confocal microscope system (Heidelberg Engineering, Heidelberg, Germany). As an immersion fluid, carbomer eye gel (Alcon) was topically administered to the murine corneas before scanning. The corneas were exposed and the objective lens (Heidelberg Engineering) was carefully contacted after anesthetization, followed by scanning the central and peripheral corneas.

### Optical Coherence Tomography (OCT) Examination

The cross-section images of the cornea or retina were taken, and the corneal or retinal thickness was noted using the OCT system (Optovue, Fremont, CA, USA). The mice were anesthetized before the examination.

### Fundus Imaging

Fundus images were obtained using an OPTOPROBE system (China). The mice were anesthetized, and tropicamide phenylephrine eye drops were used to dilate the pupils. Fundus images of each eye were captured, centered on the optic nerve head.

### H&E Staining

The mice were killed, and the eyeballs were fixed in 4% paraformaldehyde (Sigma-Aldrich). Subsequently, the corneal tissue was embedded in paraffin and sliced. Sections were stained with hematoxylin (Auragene, Hunan, China) for 5 min, washed with PBS, differentiated by 1% hydrochloric acid alcohol solution, and washed with PBS until they turned blue. The sections were dyed using eosin (Auragene) for 1.5 min, rinsed with PBS, dehydrated with gradient alcohol concentrations, and rinsed in xylene. Finally, the sections were sealed with neutral gum and observed under a light microscope (Eclipse E400 with DS-Fi1, Nikon, Melville, New York).

### Masson Staining

The Masson Trichrome Staining Kit (Jiancheng Bioengineering Institute, Nanjing, China) was employed in this study. The sections were stained with Wiegert's iron hematoxylin, differentiated in an acid ethanol solution, and stained with Masson blue solution, followed by washing with DDW. Subsequently, the sections were stained with Lechon red magenta and washed with a phosphomolybdic acid solution before staining in a blue aniline solution. This was followed by gradient alcohol dehydration, xylene transparency, neutral gum sealing, and observation under a light microscope (Nikon).

### Quantitative Real-Time PCR (qPCR)

Murine corneas were harvested, and two corneas were pooled into one tube. Total RNA was extracted using the RNeasy Mini Kit (QIAGEN, Duesseldorf, Germany) and reverse transcribed to cDNA by the Primescript First-Strand cDNA Synthesis kit (TaKaRa, Shiga, Japan). Quantitative real-time polymerase chain reaction (qRT-PCR) was performed via a Real-Time PCR system (the StepOne Plus Real-Time PCR detection system; Applied Biosystems, Darmstadt, Germany) with the SYBR TaqKit (TaKaRa). The primers used in this study are listed in Table 3. The real-time PCR data were exported as a file and analyzed using the comparative cycle threshold (Ct) method, in brief, with the following formula:

$$\begin{aligned} \Delta \Delta {\text{Ct}} & = \left( {{\text{Ct }}\;{\text{target}}\;{\text{ gene}} - {\text{Ct}}\;{\text{ endogenous }}\;{\text{reference}}\;{\text{ gene}}} \right)_{{{\text{treatment}}\;{\text{ group}}}} \\ & \quad - \left( {{\text{Ct }}\;{\text{target}}\;{\text{ gene}} - {\text{Ct}}\;{\text{ endogenous }}\;{\text{reference}}\;{\text{ gene}}} \right)_{{{\text{control}}\;{\text{ group}}}} \\ \end{aligned}$$

β-actin was normalized as an endogenous reference.

**Table 3:** Primers used for qRT-PCR

Gene	Forward primer sequence (5'-3')	Reverse primer sequence (5'-3')
β-actin	GCCCTGAAAGCTACCCAAGT	AGGAATCCTTCTGACCCATGC
IL-1β	GGGCCTCAAAGGAAAGAATC	TACCAGTTGGGGAACTCTGC
α-SMA	CCCAAAGCTAACCGGGAGAAG	CCAGAATCCAACACGATGCC
Fibronectin	GTGGCTGCCTTCAACTTCTC	TTGCAAACCTTCAATGGTCA

### Statistical Analysis

All data are expressed as the means ± SEM. The statistical analysis was performed via GraphPad Prism 8.0 software (San Diego, California, USA). Statistical significance was evaluated by the one-way ANOVA test or the unpaired Student’s *t*-test. A *p*-value less than 0.05 was considered to be statistically significant.

## Results and Discussion

### Establishment of the Structure–Property Relationship of Cu-X MOFs Nanozymes

**Fig. 2:**
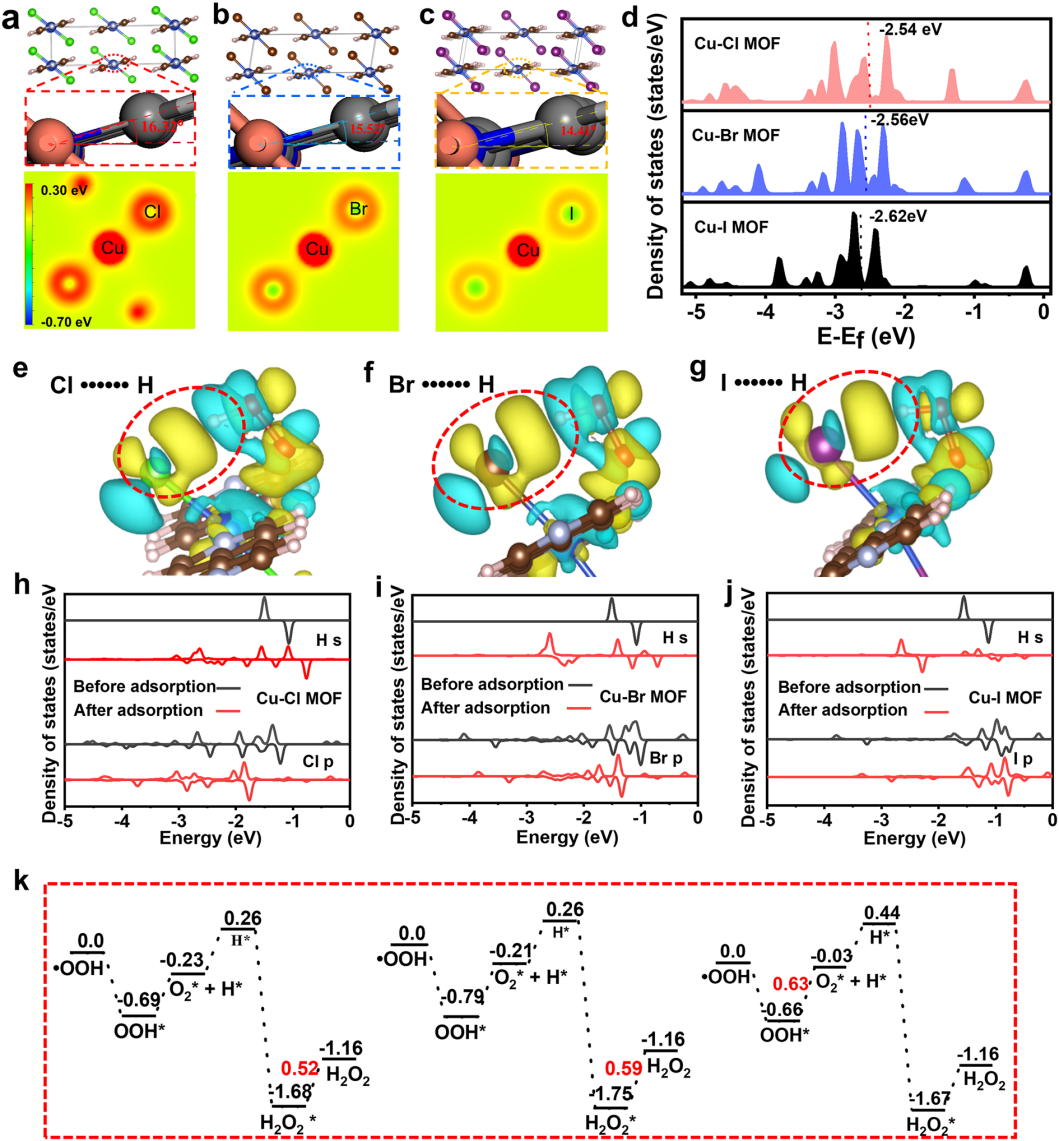
DFT studies on the electronic structures and SOD-like activity of Cu-X MOFs. **a**-**c** Dihedral angle between the copper atomic plane and 4,4’-bipyridyl plane in Cu-X MOFs and the corresponding two-dimensional Bader charge diagram. **d** The total density of states of Cu-X MOFs and the corresponding Cu 3*d* orbitals. **e**–**g** The 3D charge density difference of the Cu-X MOFs and **h**-**j** corresponding X *p* and H *s* orbitals. **k** Calculated reaction energy profiles for •OOH decomposition on the Cu-X MOFs (001) surfaces. (Unit: eV)

To test and verify the feasibility of our strategy, we first performed DFT calculations on the Cu-X MOFs crystal configuration and the electronic structure of the Cu nodes for different halogen atoms. According to the cell structures in Fig. 2a-c, the dihedral angles between the copper atomic plane and 4,4’-bipyridyl plane in Cu–Cl MOF, Cu-Br MOF, and Cu-I MOF are 16.32°, 15.52°, and 14.41°, respectively. This result indicates that the interaction order between X (Cl, Br, I) and its nearest neighboring layer Cu nodes in Cu-X coordination is Cu–Cl > Cu–Br > Cu–I. Therefore, the magnitudes of the dihedral angles between the respective planes of Cu atoms and linker benzene rings with different halogen coordination show a similar trend. To further understand the differences within the conventional cell of Cu-X MOFs with various halogens, we analyzed the Bader charge of Cu-X coordination. As shown in Fig. 2a–c, the ability of the halogen atoms to gain electrons tends to decrease as the atomic number increases. The halogen atoms in Cu-X MOFs gain 0.58, 0.33, and 0.23 |e|, and the corresponding Cu atoms lose 0.82, 0.62, and 0.19 |e|, respectively (Fig. S1). It is suggested that the ability of halogen atoms to regulate the electronic structure of Cu sites decreases with increasing atomic number.

In natural metalloproteinase-mediated reactions, the interaction of the active metal center with the substrate is an essential factor in determining the reaction rate [31]. The properties of this interaction are determined by matching the front orbitals of the reactants to the front orbitals of the active metal center. The essential behavior of catalyst-mediated reactions with transition metal active sites is reactant-induced electron rearrangement in the unfilled *d* orbitals (part of the front orbital component) of the transition metal [32]. On this basis, we further calculated the density of states (DOS) of the Cu-X MOFs for different halogen-coordinated Cu nodes using conventional cells. Figure 2d shows that the DOS of Cu near the Fermi energy level is mainly contributed by the Cu 3*d* orbitals, implying that Cu is more likely to be a site for substrate molecule adsorption. In addition, the *d* band centers of the Cu–Cl MOF, Cu-Br MOF, and Cu-I MOF models are − 2.54, − 2.56, and − 2.62 eV, respectively, indicating different bonding abilities between the catalyst and the adsorbed molecule [32, 33]. Thus, the modulation of the *d* orbital electronic structure of Cu by halogen atoms offers the possibility of modulating the reactivity of Cu-X MOFs.

Based on the example of simulated natural SOD, we further discuss halogen’s role in regulating the Cu-X MOFs’s catalytic activity. As •O_2_^−^ is a Brønsted base with p*K*_b_ = 9.12, it readily traps a proton from water to form •OOH and OH^−^, as shown in Eq. 6:

6 $$\cdot {\text{O}}_{2}^{ - } + {\text{H}}_{{2}} {\text{O}} = \cdot {\text{OOH}} + {\text{OH}}^{ - }$$

As shown in Fig. 2e–g, •OOH possesses a quite similar adsorption conformation on the Cu-X MOFs slabs. The three-dimensional diagram of the differential charges shows that in addition to the electron transfer between Cu and •OOH, there is also a significant charge transfer between •OOH and X. Notably, it is evident from the two-dimensional differential charge diagram that the electron transfer of *X* with •OOH is significantly more robust than that of Cu with •OOH (Fig. S2). The order of magnitude of electron transfer between X and •OOH is Cl••••••H > Br••••••H > I••••••H. This result indicates that X plays a vital role in the adsorption of •OOH and the subsequent reaction. Moreover, it also demonstrates the feasibility of modifying the Cu active site by using halogen atoms to regulate its activity. To further elucidate the interaction of Cu-X MOFs involved in the reaction with •OOH, we investigated the DOS of *s* orbitals of H and *p* orbitals of X before and after •OOH adsorption on Cu-X MOFs. As shown in Fig. 2h-j, the energy levels of the H's α-spin *s* orbital and the X *p* orbital in •OOH are well matched, leading to a significant cleavage of the corresponding orbital after adsorption. After •OOH adsorption, the highest occupied orbital energy levels of Cl, Br, and I coordinated to the Cu node decreased by 0.53, 0.34, and 0.01 eV, respectively, and their order of strength is that the Cl••••••H interaction is the strongest, followed by Br••••••H, and the interaction with I••••••H is the weakest. This is consistent with the numerical results for the X–H distance calculated in Fig. S3. In contrast, the *d* orbitals of Cu and the molecular orbitals of •OOH undergo different degrees of cleavage when •OOH is adsorbed on sites with different halogen coordination (Fig. S4), indicating that there are interactions of different strengths between •OOH and Cu sites. The distance between Cu and O for different halogen coordination also confirmed these results. The lengths of O and Cu-X (X = Cl, Br, I) are 2.49, 2.47, and 2.33 Å, respectively (Fig. S3), implying that the MOF with the most robust interaction with O is Cu-I, followed by Cu-Br, while Cu–Cl has the weakest interaction. It should be noted that the interaction between •OOH and Cu-X in adsorption is the interaction of the orbitals of •OOH with the X and Cu orbitals in Cu-X MOFs, within which are contained the orbital interactions between H and X as well as those between Cu and O.

One of the two main mechanisms simulating the catalytic process of SOD is the synergistic involvement of multiple sites and the involvement of elements with multiple sites, such as Au, Pd, Pt [34]. Their surfaces adsorb multiple •OOH molecules simultaneously, and the adjacent ones undergo reshooting, thus forming H_2_O_2_ and O_2_. The other major catalytic mechanism involves only a single active site. Based on the principle of SOD and a previously reported thermodynamic model (Fig. S5), Gao et al. developed a conduction band minimum (CBM)-mediated single active site catalytic mechanism [35]. The CBM-mediated catalytic mechanism is as follows:

$$\cdot {\text{O}}_{2}^{ - } + {\text{Nanomaterial}} = {\text{O}}_{2} + {\text{Nanomaterial }}^{ - }$$

$$\cdot {\text{O}}_{2}^{ - } + 2{\text{H}}^{ + } + {\text{Nanomaterial }}^{ - } = {\text{H}}_{2} {\text{O}}_{2} + {\text{Nanomaterial}}$$

The intermediate is a negatively charged nanomaterial^−^, which is first reduced by one •O_2_^−^ and subsequently oxidized back to its original state by another •O_2_^−^.

To explore the evolutionary path of •OOH on the Cu-X MOFs surface, we will discuss the Cu–Cl MOF as an example. The bond lengths of Cu-Cu in the optimal Cu–Cl MOF slab were first calculated to be 8.23 and 11.14 Å (Fig. S6), which far exceeded the distance at which the redistribution of •OOH on the two Cu sites could occur. In contrast, we calculated the energy levels of the Cu–Cl MOF surface relative to the hydrogen electrode (HE) potential. Of the two, the CBM value is closer to *φ*_1_ (Fig. S7). According to Gao's energy level theory [35], the evolution of •OOH on the Cu–Cl MOF surface follows a CBM-mediated mechanism that evolves at monometallic sites. Therefore, this study investigated the effect of halogen atoms on the catalytic performance of Cu-X MOFs mimicking natural SOD using a CBM-mediated single-site mechanism. Because Cu-X MOFs belong to the same two-dimensional material family as MoS_2_ and graphene, we used the optimal (001) surface of Cu-X MOFs in the computational model (Fig. S8). The calculated adsorption energies were − 0.69, − 0.79, and − 0.66 eV, respectively (Fig. S9), indicating that the adsorption of •OOH on the (001) surfaces of Cu-X MOFs is a highly exothermic process.

To computationally verify the above mechanism, we used DFT calculations to locate the potential energy distribution of the key intermediate and transition state structures of •OOH evolution on the Cu-X MOFs surface. First, •OOH passes hydrogen to the halogen, reducing the Cu-X MOFs by forming an intermediate hydrogen which oxidizes the Cu-X MOFs back to their original state. The above analysis shows that the adsorption energy of •OOH on the Cu-X MOFs surface mainly originates from two factors. One is the interaction of halogens with •OOH, the order of strength of which is Cu–Cl MOF > Cu–Br MOF > Cu–I MOF. The other is the interaction of Cu with •OOH, the order of strength of which is Cu-I MOF > Cu–Br MOF > Cu–Cl MOF. The opposite order of the two interactions leads to the strongest adsorption energy of the Cu-Br MOF for •OOH, which results in a higher energy barrier than that of the Cu–Cl MOF in the dehydrogenation of •OOH. In contrast, the interaction between X and •OOH was more robust than that between Cu and •OOH (Fig. S1). Therefore, the Cu–Cl MOF shows moderate adsorption of •OOH. Meanwhile, the energy barriers of the rate-determining step of the Cu–Cl MOF-catalyzed reaction are 0.52, 0.59, and 0.63 eV, respectively (Fig. 2k). Consequently, the order of the SOD-like activity obtained from these energy calculations is Cu–Cl MOF > Cu–Br MOF > Cu–I MOF. These results theoretically explain the modulation of the Cu-X MOFs catalytic activity by halogen atom coordination of the Cu active centers.

### Synthesis and Structural Characterization of the Cu-X MOFs Nanozymes

**Fig. 3:**
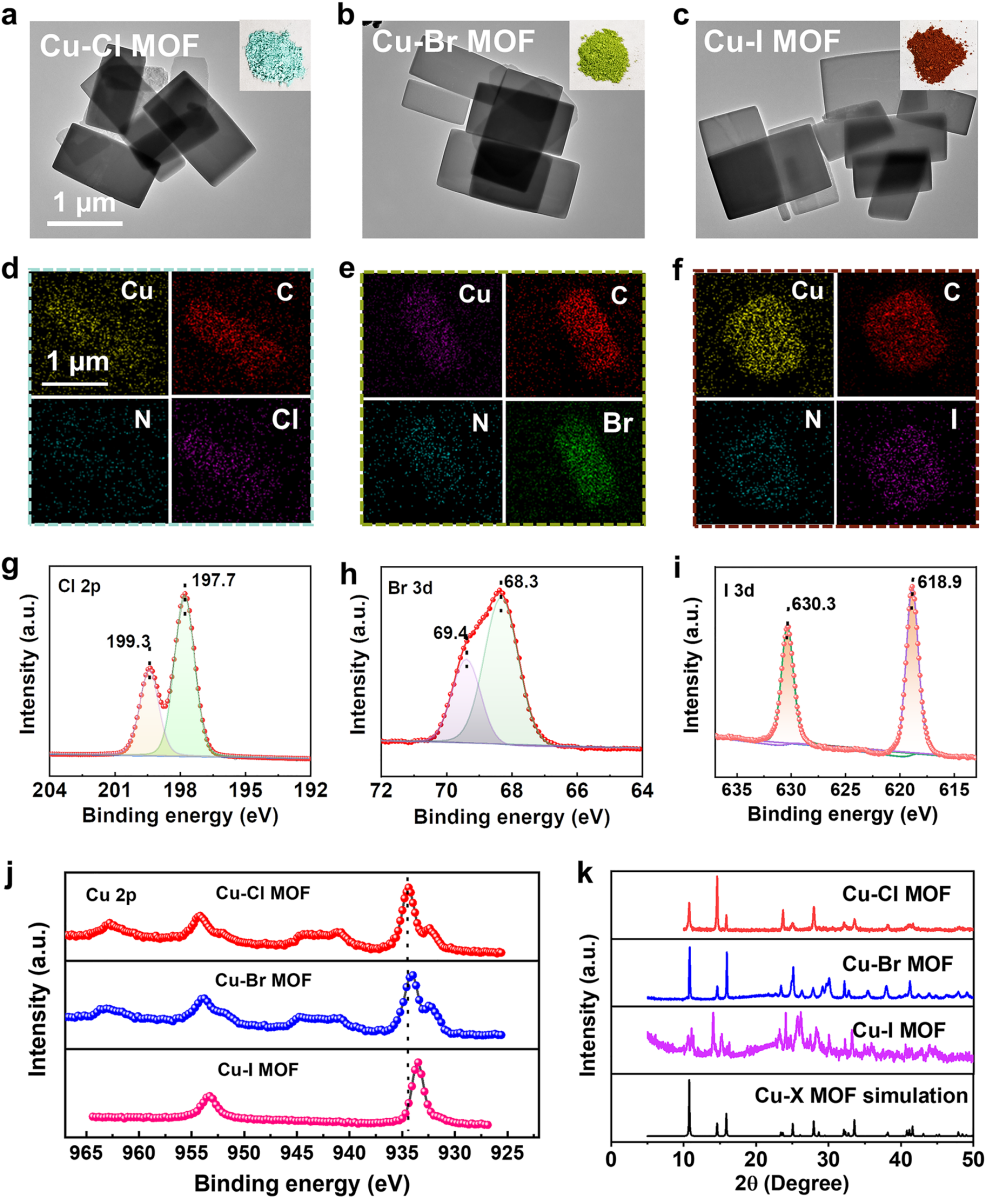
Characterization of Cu-X MOFs**. a**-**c** TEM images of Cu-X MOFs. Inset: photos of powder samples; and **d**-**f** the corresponding EDS mapping. **g**-**j** XPS spectrum: **g** Cl 2*p,* **h** Br 3*d,* **i** I 3*d,* **j** Cu 2*p*. **h** XRD patterns of Cu-X MOFs

A series of Cu-X MOFs with tunable coordination microenvironments were prepared to verify our theory. The morphology and structure of Cu-X MOFs were characterized using various techniques. Representative transmission electron microscopy (TEM) images show that the Cu-X MOFs are a morphologically homogeneous two-dimensional nanosheets (Fig. 3a–f). The X-ray diffraction (XRD) patterns show that the experimentally synthesized samples had characteristic peaks similar to those of the simulated Cu-X MOFs (Fig. 3k) [30]. Furthermore, the surface composition of the Cu-X MOFs was analyzed by X-ray photoelectron spectroscopy (XPS) (Figs. 3g–j and S10). As shown in Fig. 3g–i, the very sharp characteristic peaks of Cl 2*p*, Br 3*d*, and I 3*d* in the high-resolution XPS spectra imply the presence of large amounts of Cl, Br, and I on the Cu-X MOFs surface [23]. The detailed XPS study of Cu 2*p* in Fig. 3j shows that halogen coordination has different binding energies, indicating modulation of the electronic structure of the metal nodes by the halogens. Generally, the loss of electrons from a given element shifts its binding energy in the high-field direction [36]. The order of the magnitude of the binding energy of Cu 2*p* for different halogen coordination is Cu–Cl MOF > Cu–Br MOF > Cu–I MOF, which is consistent with the trend of electrons gained by DFT calculations. These results indicate that Cu-X MOFs with different halogen-coordinated Cu nodes were successfully constructed.

### SOD- and CAT-Like Activities of Cu-X MOFs Nanozymes

**Fig. 4:**
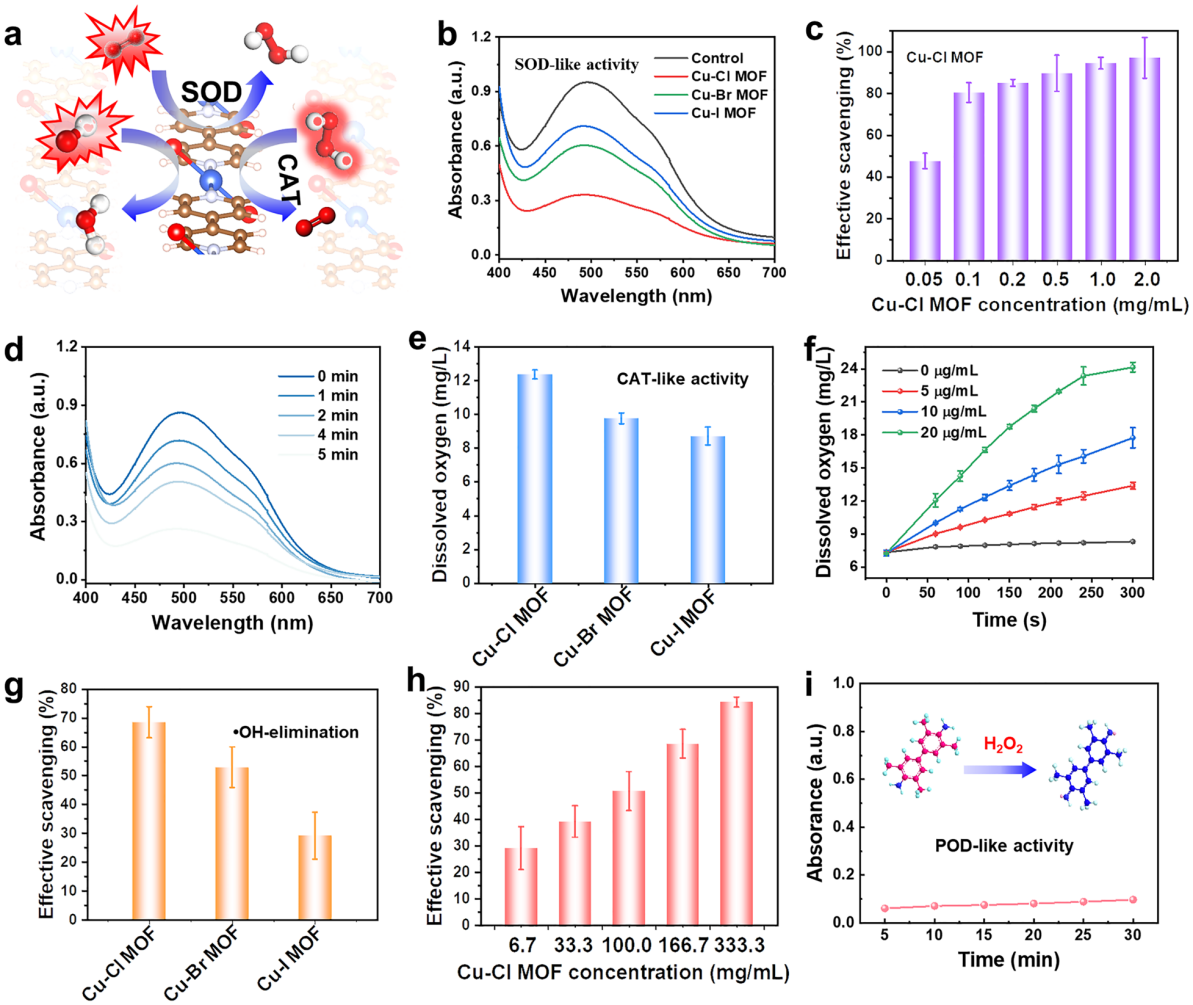
ROS scavenging and multienzyme-like antioxidative activity of Cu-X MOFs. **a** Schematic illustration of SOD- and CAT-like characteristics of Cu-X MOF. **b** Absorption spectra of iodonitrotetrazolium chloride after reaction with •O_2_^−^ in Cu-X MOFs. **c** The removal efficiency of •O_2_^−^ with different masses of Cu–X MOF. **d** Absorption spectra of iodonitrotetrazolium chloride after reaction with •O_2_^−^ for different times. **e** The activity of Cu–Cl MOFs to catalyze the decomposition of H_2_O_2_ to produce O_2_. **f** Kinetic curves of O_2_ production at different concentrations of H_2_O_2_ catalyzed by Cu–Cl MOFs. **g** The activity of Cu-X MOFs for •OH elimination. **h** The activity of •OH removal by Cu–Cl MOF at different concentrations. **i** The peroxidase-like activity of Cu–Cl MOF

Iodonitrotetrazolium chloride (INT) was used as a probe to explore the SOD-like activity of the Cu-X MOFs. INT is a sensitive superoxide indicator with strong absorption at 505 nm when reduced by •O_2_^−^ (Fig. S11). As shown in Fig. 4b, in the absence of the Cu-X MOFs, INT was reduced by •O_2_^−^ with a significant absorption peak at 505 nm. However, in the presence of Cu-X MOFs, •O_2_^−^ is decomposed by Cu-X MOFs, inhibiting the reduction of INT by •O_2_^−^. The order of the SOD-like activity is Cu–I MOF < Cu–Br MOF < Cu–Cl MOF (Fig. 4a-b), which is consistent with the calculated results. To exclude the effect of the specific surface area on the activity, we measured the nitrogen adsorption–desorption isotherms of the Cu-X MOFs. The specific surface areas were 18.51 (Cu–Cl MOF), 20.67 (Cu–Br MOF), and 19.53 m^2^ g^−1^ (Cu-I MOF) (Figs. S12 and S13), indicating that the specific surface area was not the main reason for the difference in the SOD-like activity of Cu-X MOFs. This is further evidence that the modulation of halogens causes a difference in Cu-X MOFs activity. Besides, the specific activities of different nanozymes were evaluated to quantitatively reflect the enzyme-like activities. The specific activity of Cu–Cl MOF is 15, 29, and 40 times higher than those reported for CuWK (WK: wool keratin), CuSF (SF: silk fibroin), and commercial Cu_2_O (Table S1), respectively.

To further explore the use of Cu-X MOFs nanozymes in antioxidant applications, the ability of Cu-X MOFs to remove other reactive oxygen species (ROS), such as H_2_O_2_ and •OH, was tested (Figs. 4e-h, S14 and S15). The Cu–Cl MOF is superior to the Cu–Br MOF and Cu–I MOF in removing H_2_O_2_ and •OH. A distinguishing feature of artificial enzymes is their high thermal stability, which is necessary for their practical applications. Therefore, we compared the catalytic activity of Cu–Cl MOF nanozymes stored for different periods. The catalytic activity of the Cu–Cl MOFs was not affected by increasing storage time; significantly, the activity was hardly reduced after storage for 90 days (Fig. S16). This indicates that the Cu–Cl MOF has excellent stability at room temperature.

### In Vitro Cytotoxicity and ROS-Scavenging Activities of Cu–Cl MOF

**Fig. 5:**
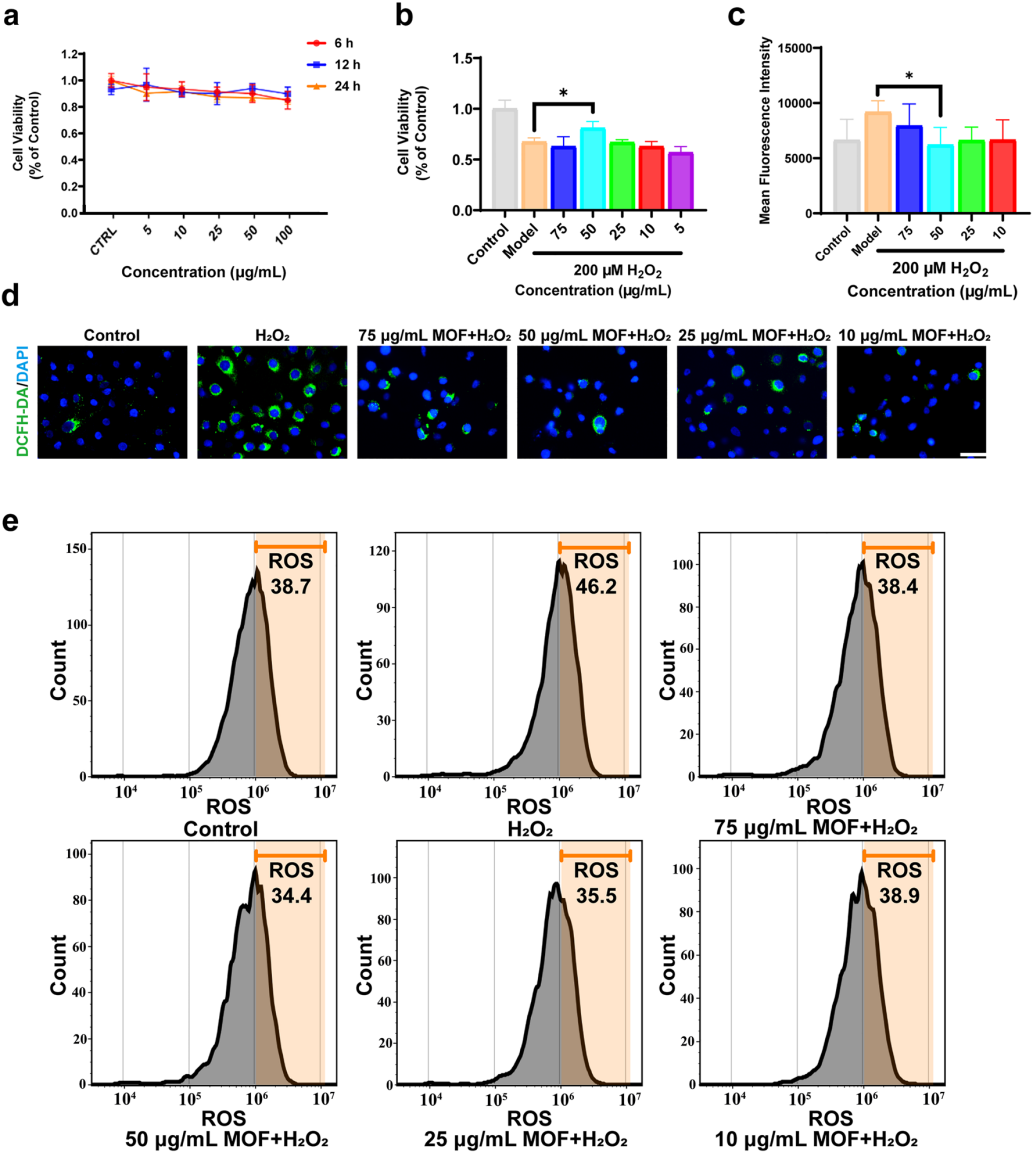
In vitro cytotoxicity and ROS-scavenging activities of Cu–Cl MOF. **a** Cell viability of HCE cells incubated with different concentrations for Cu–Cl MOF in 6, 12, and 24 h via CCK-8 assays. **b** Cell viability of HCE cells incubation with the 200 µM H_2_O_2_ and different concentrations of Cu–Cl MOF. **c, d** Quantitative statistics of fluorescence intensity and representative fluorescence images for intracellular ROS detection via DCFH-DA staining, *Scale bar*: 100 μm. **e** Flow cytometry tests of ROS levels in HCE cells via DCFH-DA staining. Data shown as the mean ± SEM. *P < 0.05

Chemical burns have been reported to cause oxidative stress from large amounts of ROS on the ocular surface, disrupting the ocular surface’s antioxidant capacity and ultimately leading to cell death and inflammation. In addition, endogenous antioxidant drugs have been used to have therapeutic effects on various ocular diseases, such as neuroprotective effects on glaucoma, retinitis pigmentosa, and optic neuritis [37]. Taking advantage of the high CAT- and SOD-like activities of optimal Cu–Cl MOF nanozymes, which could catalytically eliminate ROS in physiological environments, we subsequently evaluated the Cu–Cl MOF nanozymes as a potential antioxidant drug in the treatment of ocular chemical burn diseases. First, to test the biocompatibility of the Cu–Cl MOF nanozymes, cytotoxicity tests were performed via CCK-8 assay in HCE cells. HCE cells are the outermost cellular layer of the cornea, which first contacts the drug. As shown in Fig. 5a, Cu–Cl MOF at concentrations ranging from 5 to 100 μg mL^−1^ showed no significant cytotoxicity to HCE cells when cocultured from 6 to 24 h. Meanwhile, the effects of Cu–Cl MOF to protect the cell from damage by oxidative stress were further explored using the oxidative stress model established by incubating HCE cells with 200 µM H_2_O_2_ for 6 h. Without being treated with Cu–Cl MOF, HCE cell viability was significantly decreased due to H_2_O_2_ treatment. Intriguingly, with the increased Cu–Cl MOF concentration, the cell viability showed a Gaussian-like distribution. This result may be due to the limited antioxidant activity of Cu–Cl MOF at low concentrations. At the same time, material toxicity was the main reason for the decrease in cell viability at high concentrations (Fig. 5b).

To visualize the process of reduction of intracellular oxidative stress by Cu–Cl MOF, the ROS probe 2,7-dichlorofluorescein diacetate (DCFH-DA) was employed. After incubation with H_2_O_2_ and different concentrations of Cu–Cl MOF, the intracellular fluorescence of DCFH-DA was observed (Fig. 5d), and the fluorescence intensity was quantified (Fig. 5c). The ROS level represented by DCFH-DA in the H_2_O_2_ group was strikingly higher than that in the control group. By contrast, Cu–Cl MOF decreased the ROS level effectively, especially at the concentration of 50 μg mL^−1^ Cu–Cl MOF. The cellular ROS levels were further verified by DCFH-DA assay through flow cytometer analysis. As shown in Fig. 5e, H_2_O_2_ triggered to achieve 46.2% ROS positivity in HCE cells compared to 38.7% in the control group. However, in the DCFH-DA fluorescence staining, the Cu–Cl MOF decreased the percentage of ROS-positive cells to 38.4%, 34.4%, 35.5%, and 38.9%, corresponding to the Cu–Cl MOF concentrations of 75, 50, 25, and 10 μg mL^−1^. Altogether, Cu–Cl MOF scavenged the ROS in the HCE cells and protected the cells against oxidative stress. The antioxidant function was derived from the excellent muti-enzyme-like activities of Cu–Cl MOF that promotes biological redox reactions.

### Potential Antioxidant and Antiapoptotic Mechanisms of Cu–Cl MOF

**Fig. 6:**
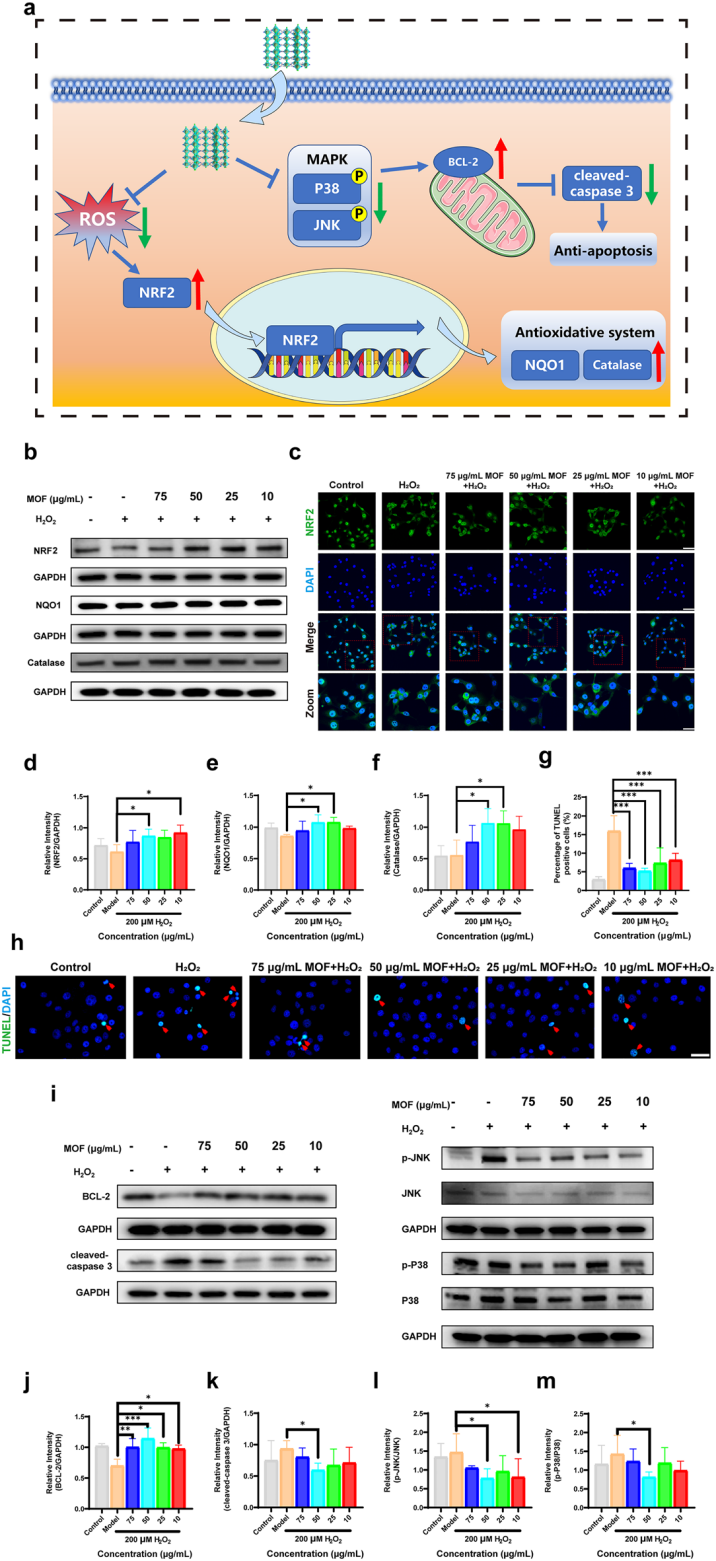
Potential antioxidant and antiapoptotic mechanisms of Cu–Cl MOF. **a** Scheme of antioxidant and antiapoptotic mechanisms of Cu–Cl MOF. **b** Protein levels of NRF2, NQO1, and catalase in HCE cells determined by Western blotting, using GAPDH as the loading control. **c** Representative fluorescence images of HCE cells incubated with 200 µM H_2_O_2_ and different concentrations of Cu–Cl MOF via NRF2 immunofluorescent staining, *Scale bar*: 100 μm (Zoom indicates the NRF2 position, *Zoom scale bar*: 50 μm). **d**-**f** Quantitative statistics for the NRF2, NQO1, and catalase protein. **g, h** Quantitative statistics of TUNEL positive cells and representative fluorescence images of TUNEL staining in HCE cells, *Scale bar*: 50 μm. **i** Protein levels of BCL-2, cleaved-caspase 3, p-JNK, p-P38, JNK, and P38 in HCE cells determined by Western blotting, GAPDH was used as the loading control. **j**-**m** Quantitative statistics for the protein of BCL-2, cleaved-caspase 3, and the ratio of p-JNK to JNK, and p-P38 to P38. Data are shown the mean ± SEM. *P < 0.05, **P < 0.01, ***P < 0.001

Oxidative stress disturbs the oxidation–reduction balance due to the excess oxidants over the antioxidant capability of cells that activate the signaling cascade within the cell [38]. As we all know, the NF-E2-related factor 2 (NRF2) system is an evolutionarily conserved defense mechanism against oxidative and xenobiotic stress [39]. To further investigate the potential mechanism by which Cu–Cl MOF inhibited oxidative stress in HCE cells, we examined the levels of critical NRF2 proteins. The results showed that Cu–Cl MOF up-regulated NFR2 expression compared with the H_2_O_2_-stimulated group, especially at a concentration of 50 μg mL^−1^ (Fig. 6b, d). This result was also confirmed by immunofluorescence staining. The nuclear staining of NRF2 was enhanced by treatment with Cu–Cl MOF, which activated the expression of the following antioxidant factors expressions. The nuclear protein level of NRF2 was further confirmed (Figs. 6c and S17). Specifically, the levels of some enzymes, such as NAD(P)H: quinine oxidoreductase 1 (NQO1) and catalase, were increased (Fig. 6b, e, f). Therefore, Cu–Cl MOF may activate the NRF2 pathway to balance biological oxidation–reduction in HCE cells.

On the other hand, excess ROS induce cell death, especially apoptosis [40]. Therefore, it is essential to study the apoptotic process of HCE cells in the experiment to gain an in-depth understanding of the antioxidant effect of Cu–Cl MOF. From TUNEL staining in Fig. 6g, h, it can be seen that H_2_O_2_ induced apoptosis in HCE cells, while Cu–Cl MOF counteracted the apoptotic process. The BCL-2 and caspase 3 are crucial factors in the execution of apoptosis for directing the apoptosis level in cells [41]. BCL-2 protects cells from apoptosis induced by endogenic stimuli, and a series of events promotes various apoptotic mediators that ultimately activate caspases [42]. Cu–Cl MOF up-regulated the level of antiapoptotic protein BCL-2 and inhibited the apoptotic marker caspase 3 compared to the H_2_O_2_ group (Fig. 6i-k).

Subsequently, further research focused on the pathway to adapt the alteration of apoptosis level. JNK and P38 are essential proteins in the MAPK pathway, and the stress-activated JNK and p38 MAPK play vital roles in balancing cell survival and death in response to oxidative stress [43]. The phosphorylation levels of JNK and P38 (active form) were significantly down-regulated by the treatment of Cu–Cl MOF compared to the H_2_O_2_ group, which indicated that Cu–Cl MOF inhibited apoptosis by decreasing the activation of JNK and p38 MAPK (Fig. 6i, l, m). In summary, Cu–Cl MOF balances the ROS level through the NRF2 pathway and controls apoptosis via the JNK and p38 MAPK pathways.

### Cu–Cl MOF Alleviated Corneal Damage in the Alkali Burn Animal Model

**Fig. 7:**
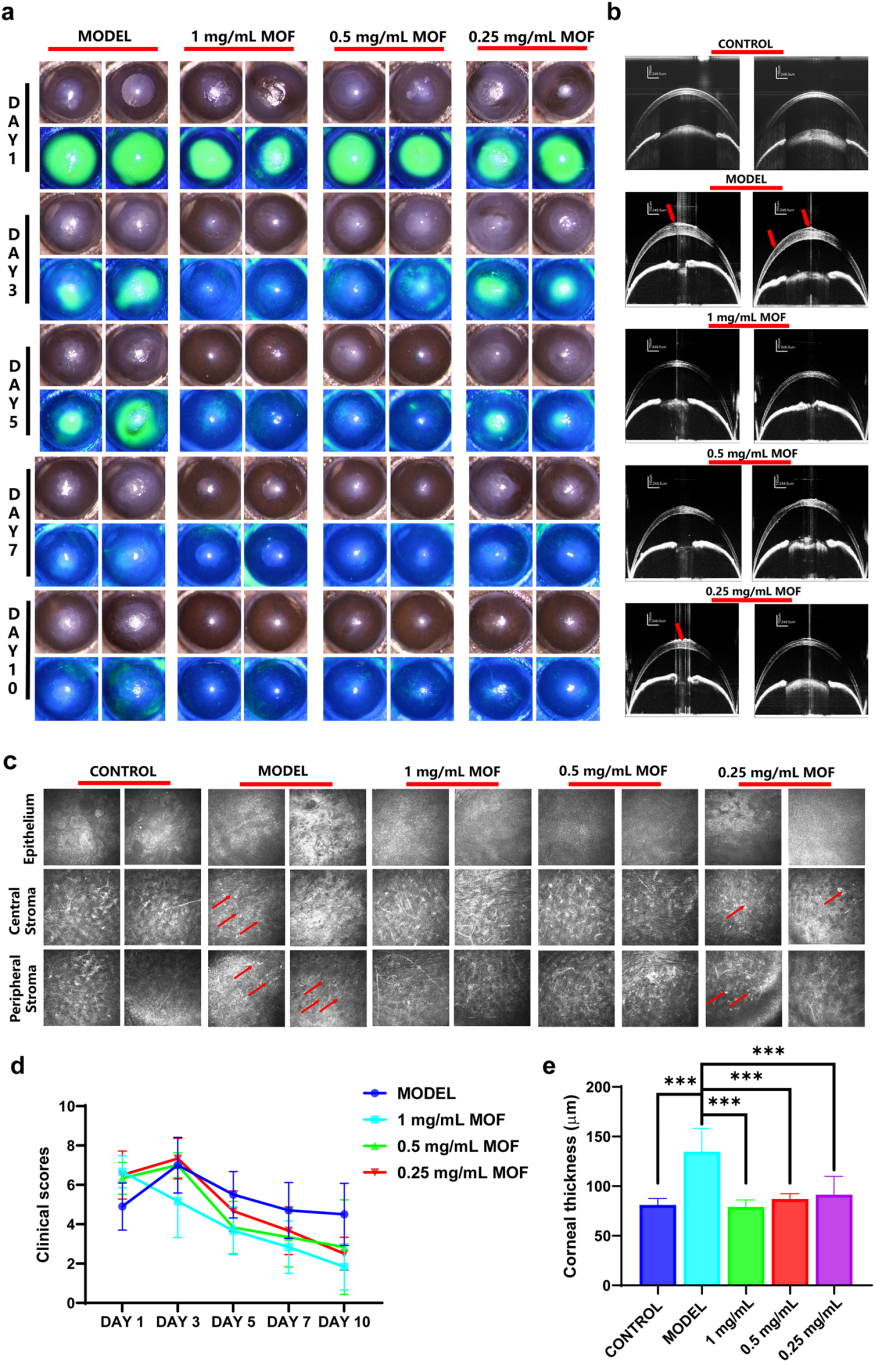
Cu–Cl MOF alleviated corneal damage in the alkali burn animal model. **a** Broad beam lighting and fluorescein staining images of ocular surface treated with different concentrations of Cu–Cl MOF after alkali burn on days 1, 3, 5, 7, and 10. **b** Representative OCT images of the anterior segment for the ocular surface (red arrow indicates the corneal epithelium defects or irregular). **c** In vivo confocal microscopy images of the corneal epithelial, central, and peripheral corneal stroma (red arrow indicates leukocyte infiltration). **d** Clinical score for the murine corneas according to slit lamp images. **e** Quantitative statistics of the corneal thickness measured and analyzed by OCT. Data are shown as the mean ± SEM. ***P < 0.001

To further evaluate the potential of Cu–Cl MOF in treating ocular chemical eye burns, a model of corneal burns was established using sodium hydroxide in adult C57BL/6 mice. The clinical score was collected and analyzed according to the Visual Scoring System for Murine Cornea in Table 2. As shown in Figs. 7a and S18, the epithelial defects were immediately severe in all mice, as shown by sodium fluorescein staining. A gradual recovery was seen until Day 7 in the model group. At the same time, the administration of Cu–Cl MOF eye drops accelerated the healing of corneal epithelium, mainly applied at a concentration of 0.5 mg mL^−1^ Cu–Cl MOF eye drops, which almost recovered on Day 3. Alkali resulted in severe inflammation and caused the cloudiness of the cornea. In the observation using the slit lamp, the cloudy cornea peaked on Day 3 in the Model group and lasted until the end of the study. Cu–Cl MOF effectively reduced the progression of the cloudiness in the cornea according to the clinical scores, hinting at the excellent suppression of corneal inflammation (Fig. 7d). This proves that MOF inhibited oxidative stress, further controlling the corneal inflammation.

The effect of Cu–Cl MOF on corneal microanatomy and integrity after chemical burns in living mice was further investigated by an optical coherence tomography (OCT) imaging system. As we all know, alkali causes severe corneal edema and opacity as a high reflection of inflammatory OCT features. Meanwhile, epithelial defects and irregularity could be found (Fig. 7b). Cu–Cl MOF resulted in much-reduced exudation or edema in contrast with the model group. In addition, chemical factors that induce inflammation in the ocular surface trigger corneal edema and further influence the corneal transparency. Since Cu–Cl MOF scavenges harmful free radicals at an early stage, it helps to restore corneal transparency. In addition, edema of the cornea increases the thickness of the cornea, and the severity of corneal alkali burns can be determined by observing the thickness of the cornea. As shown in Fig. 7e, Cu–Cl MOF eye drops significantly controlled the corneal thickness and avoided corneal edema compared to the control group.

In vivo confocal microscopy (IVCM) is a noninvasive examination technique that allows the observation of physiological or pathological changes in the cornea at a cellular level in an alkali burn [44]. As shown in Fig. 7c, the alkali destructed the corneal epithelium, and recruited leukocytes (with high reflection in the IVCM) infiltrating the central or peripheral corneal stroma, leading to a disorderly structure. MOF eye drop administration protected the corneal epithelium and stroma, keeping the corneal stroma cells at rest. However, at a 0.25 mg mL^−1^ concentration, few inflammatory cells were observed in the corneal stroma, probably due to the low concentration, thus limiting the anti-inflammatory effects. Generally, Cu–Cl MOF eye drops presented an effective therapeutic in treating corneal alkali burns based on clinical ophthalmic analysis in animals.

### Cu–Cl MOF Reduced Corneal Inflammation and Fibrosis After Alkali Burn

**Fig. 8:**
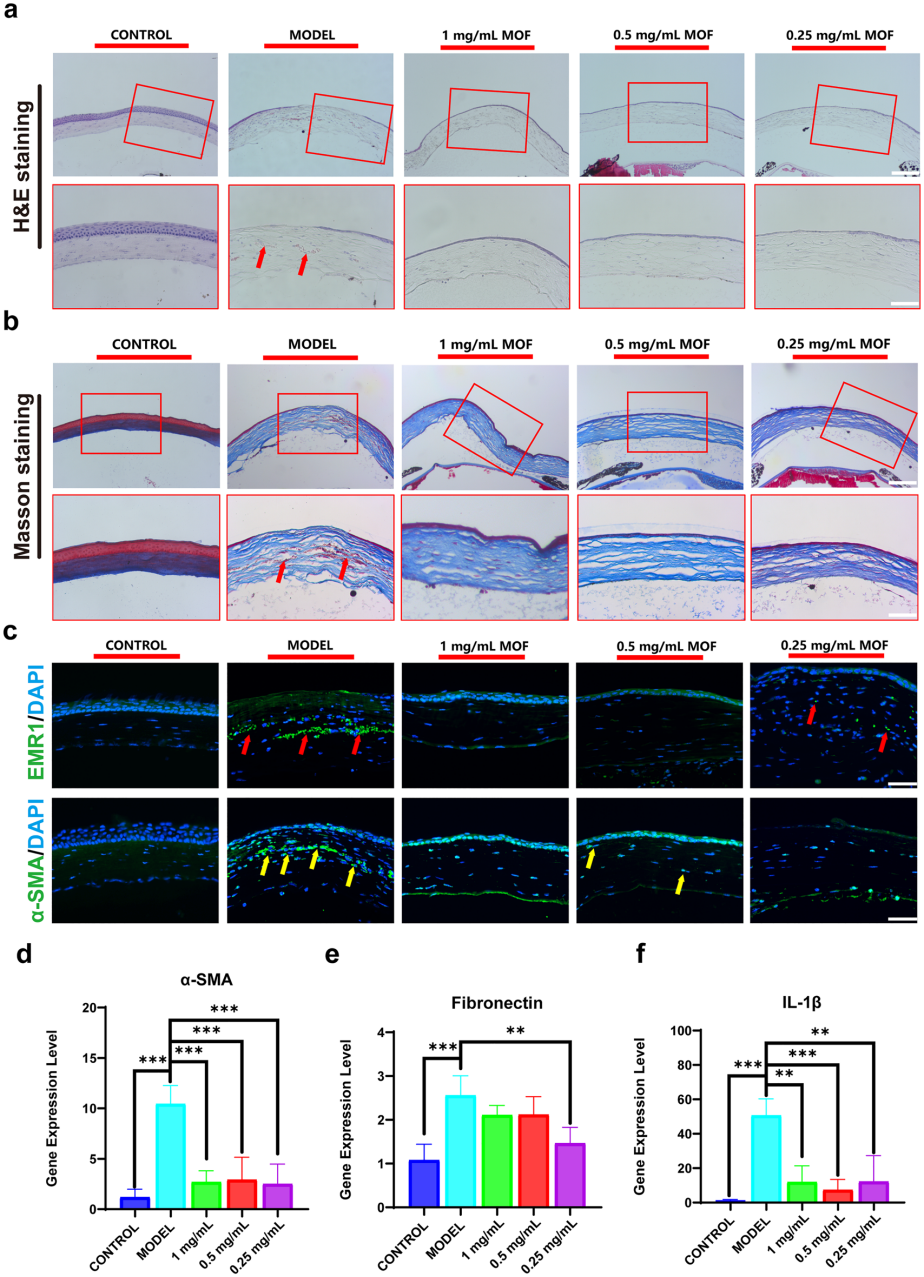
Cu–Cl MOF reduced corneal inflammation and fibrosis after alkali burn. **a** H&E staining showing central structures of the cornea, *Scale bar*: 100 μm (red rectangles zoom the cornea and red arrow indicate the inflammatory cells, *Zoom scale bar*: 50 μm). **b** Masson staining showing the collagen arrangement of the cornea, *Scale bar*: 100 μm (red rectangles zoom the cornea and red arrow indicate the area of the disordered structure, *Zoom scale bar*: 50 μm). **c** EMR1 staining showing the position and number of macrophages (red arrow) and α-SMA staining showing the area of fibrosis and scar-formation (yellow arrow), *Scale bar*: 50 μm. **d-f** Quantitative statistics for the gene expression levels of α-SMA, fibronectin, and IL-1β. Data are shown as the mean ± SEM. *P < 0.05, **P < 0.01, ***P < 0.001

Histological analysis was conducted to clarify the detailed pathological process in corneal alkali burn treatment. H&E staining (Fig. 8a) showed a significant infiltration of leukocytes in the corneal stroma, tissue fibrosis deforming the cornea, and a significant defect in the corneal epithelium in the model group. Although structural disorders were observed in Cu–Cl MOF eye drops at a low concentration of 0.25 mg mL^−1^, showing the presence of epithelial injury. However, Cu–Cl MOF at concentrations of 1 and 0.5 mg mL^−1^ was beneficial in renewing the typical structures of corneas that were burned by the alkali burn. Alkali has been reported to disrupt the stromal collagenous fibrillary arrangement [45]. This is mainly due to the replacement of collagen fibers by *α*-actin fibers, protein sediments, and cell infiltrates. Masson staining was performed to determine the collagen arrangement. The corneal structure recovered to a stable and complete lamellar structure after applying 0.5 and 1 mg mL^−1^ Cu–Cl MOF eye drops (Fig. 8b). Collagen fibers were more disordered in the model group. Therefore, MOF reduced oxidative stress inflammation and further hindered the cells from reconstructing the cornea with irregular healing.

In response to the injury caused by corneal alkali burn, macrophages migrate to the inflammatory area, and macrophages remain there longer than other immune cells [46]. F4/80 is a widely used marker for macrophages, a member of the EGF-TM7 family, and includes an EGF-like module containing mucin-like hormone receptor-like 1 (EMR1) [47]. Hence, in this study, EMR1 was designed to indicate the position and quantity of macrophages. Many EMR1 labeled cells could be found within the Model group's corneal stroma, due to the severe macrophage infiltration induced by alkali burn (Fig. 8c). While 1 and 0.5 mg mL^−1^ Cu–Cl MOF eye drop effectively reduced macrophages in the cornea, α-smooth muscle actin (α-SMA) reflected myofibroblast levels. It hints at fibrosis and scarring in the tissue [48]. The α-SMA positive area was evident in the model group, and Cu–Cl MOF eye drops alleviated the intensity of α-SMA staining (Fig. 8c). It demonstrated the ability to control fibrosis and scarring in the cornea by Cu–Cl MOF eye drops, which contributed to the recovery of the transparency in the cornea. Finally, we detected the expression of the fibrosis-related genes, α-SMA, and fibronectin, and found the same trend in the α-SMA immunofluorescence staining (Fig. 8d-e). In addition to the expression of inflammatory cytokines, IL-1β was tested by the qPCR system, and it was found that Cu–Cl MOF reduced the expression of IL-1β in the cornea in resisting the inflammation by alkali (Fig. 8f). Therefore, Cu–Cl MOF relieved the inflammation and fibrosis from alkali burns and was important in reducing corneal injury.

### Safety Analysis of Cu–Cl MOF Eye Drops in Mice

As a novel drug for ocular surface disease, the safety of the Cu–Cl MOF still requires verification. In this study, continuous administration of Cu–Cl MOF eye drops was conducted in mice three times daily (09:00 am, 3:00 pm, and 9:00 pm) for 14 days to ensure safety for ophthalmic usage. No corneal edema or clouds were observed in the slit lamp study of each group that accepted Cu–Cl MOF eye drops (Fig. S19). After applying the Cu–Cl MOF eye drops, the cornea stayed transparent, and the epithelium was smooth. Anterior segment OCT (AS-OCT) was utilized to observe the tiny changes within the live mice's cornea. The corneal structure was regular, and there was no apparent inflammation in the corneal stroma when Cu–Cl MOF was used. In addition, no significant exudation in the anterior chamber and narrowing of the chamber were found in all the mice that received Cu–Cl MOF eye drops (Fig. S20). Corneal thickness stayed normal according to the measurement and calculation by AS-OCT (Fig. S21). IVCM was further conducted to observe the changes at the cellular level in the cornea after using Cu–Cl MOF eye drops. Histological anatomic corneal structures were observed in the normal mice and each treatment group. All corneal layers maintained normal states, including the epithelium, stroma, and endothelium (Fig. S22).

Previous reports described the immune reaction by the nanoparticles in the retina, which might injure the retinal structure, further influencing the retina's function [49]. The fundus imaging and posterior segment OCT (PS-OCT) were performed to observe whether such side effects existed on the retina. As shown in the fundus imaging (Fig. S23), no noticeable change was found in the morphology of the optic disc or retinal blood vessels, as well as no significant retinal degeneration, hemorrhage, hemangioma, new blood vessels, exudation, atrophy spots, pigment disorder, retinal hiatus, or other changes to the retina were observed among the treatment groups. PS-OCT examination of the retina showed no significant difference from the normal murine retina. Each layer of the retina was arranged orderly, no difference in retinal thickness was observed, and there was no apparent retinal degeneration or detachment among the groups (Fig. S24). Meanwhile, we measured and calculated the thickness of the retina and found no significant difference between the normal mice and the mice that received Cu–Cl MOF treatment (Fig. S21b). Combined, Cu–Cl MOF is effective in relieving chemical corneal burns and has good biocompatibility for the eye, making it suitable for clinical application in ophthalmology. Although it has been reported that metal nanozymes overcome the limitations of many natural enzymes, the development of MOFs still faces significant challenges. The dissociation of metal ions and organic connectors released by MOFs may cause potential biosafety problems. Although Cu–Cl MOFs had no apparent toxic or side effects on animals in this study, viable strategies are still needed to reduce the unnecessary long-term accumulation of these agents in normal tissues and accelerate their clearance in vivo. Biodegradable/absorbable components in MOFs may be a potential solution to this problem. In future research, spatiotemporally controllable treatments will have potential applications in improving the biosafety of MOF-based agents.

## Conclusion

In the present study, a new class of Cu-X MOFs (*X* = Cl, Br, I)-based artificial enzymes with well-defined electronic and geometric structures were developed to mimic the catalytic center of highly evolved natural enzymes. Both theoretical and experimental results demonstrated that the enzyme-like activities of the developed Cu-X MOFs could be modulated by directly altering the coordination microenvironment of the Cu active center. In addition, the origin of halogen atom modulation of Cu-X MOFs activity, that is, the interaction of the auxiliary Cu active center with reactants or intermediates such as •OOH and H, was proposed for the first time. Uniquely, the interaction between the halogen and •OOH was more robust than that between •OOH and Cu, when •OOH evolved on the Cu-X MOFs surface. Moreover, we also demonstrated that Cu–Cl MOFs possess optimal SOD-like activity that could scavenge H_2_O_2_ and •OH. Excellent biocompatibility of Cu–Cl MOF was observed with HCE cells, and the potential antioxidant and antiapoptotic mechanisms of Cu–Cl MOF were realized by regulation of NRF2 and JNK or P38 MAPK. By eliminating excess reactive oxygen species from the damage sites in the cornea, Cu–Cl MOF nanozymes finished the inflammation and fibrosis in a murine model of chemical corneal burn. Our work opens new horizons for understanding the effect of the coordination environment on the enzymatic activity of nanozymes and establishing a structure–activity relationship, thereby providing a new potential strategy for treating ocular surface disease.

### Supplementary Information

Below is the link to the electronic supplementary material.

Supplementary file1 (PDF 1465 KB)

## References

[1] Bizrah M, Yusuf A, Ahmad S (2019). An update on chemical eye burns. Eye.

[2] Gu X-J, Liu X, Chen Y-Y, Zhao Y, Xu M (2016). Involvement of NADPH oxidases in alkali burn-induced corneal injury. Int. J. Mol. Med..

[3] Cejka C, Cejkova J (2015). Oxidative stress to the cornea, changes in corneal optical properties, and advances in treatment of corneal oxidative injuries. Oxidative Med. Cell. Longev..

[4] Ji S, Jiang B, Hao H, Chen Y, Dong J (2021). Matching the kinetics of natural enzymes with a single-atom iron nanozyme. Nat. Catal..

[5] Liu Q, Zhang A, Wang R, Zhang Q, Cui D (2021). A review on metal-and metal oxide-based nanozymes: Properties, mechanisms, and applications. Nano-Micro Lett..

[6] Li F, Sun H, Ren J, Zhang B, Hu X (2022). A nuclease-mimetic platinum nanozyme induces concurrent DNA platination and oxidative cleavage to overcome cancer drug resistance. Nat. Commun..

[7] Zhang Z, Zhang X, Liu B, Liu J (2017). Molecular imprinting on inorganic nanozymes for hundred-fold enzyme specificity. J. Am. Chem. Soc..

[8] Wei H, Gao L, Fan K, Liu J, He J (2021). Nanozymes: A clear definition with fuzzy edges. Nano Today.

[9] Zhang X, Cheng L, Lu Y, Tang J, Lv Q (2022). A mxene-based bionic cascaded-enzyme nanoreactor for tumor phototherapy/enzyme dynamic therapy and hypoxia-activated chemotherapy. Nano-Micro Lett..

[10] Gao L, Zhuang J, Nie L, Zhang J, Zhang Y (2007). Intrinsic peroxidase-like activity of ferromagnetic nanoparticles. Nat. Nanotechnol..

[11] Chen J, Liu Y, Cheng G, Guo J, Du S (2022). Tailored hydrogel delivering niobium carbide boosts ROS-scavenging and antimicrobial activities for diabetic wound healing. Small.

[12] Li J, Wang S, Lin X, Cao Y, Cai Z (2022). Red blood cell-mimic nanocatalyst triggering radical storm to augment cancer immunotherapy. Nano-Micro Lett..

[13] Song Y, Qu K, Zhao C, Ren J, Qu X (2010). Graphene oxide: intrinsic peroxidase catalytic activity and its application to glucose detection. Adv. Mater..

[14] Das B, Franco JL, Logan N, Balasubramanian P, Kim MI (2021). Nanozymes in point-of-care diagnosis: An emerging futuristic approach for biosensing. Nano-Micro Lett..

[15] Zeng Q, Qi X, Shi G, Zhang M, Haick H (2022). Wound dressing: from nanomaterials to diagnostic dressings and healing evaluations. ACS Nano.

[16] Jiang B, Liang M (2021). Advances in single-atom nanozymes research. Chin. J. Chem..

[17] Liu Y, Cheng Y, Zhang H, Zhou M, Yu Y (2020). Integrated cascade nanozyme catalyzes in vivo ROS scavenging for anti-inflammatory therapy. Sci. Adv..

[18] Wang D, He IW, Liu J, Jana D, Wu Y (2021). Ligand-dependent activity engineering of glutathione peroxidase-mimicking MIL-47(V) metal-organic framework nanozyme for therapy. Angew. Chem. Int. Ed..

[19] Chen M, Lang L, Chen L, Wang X, Shi C (2022). Improving in vivo uranyl removal efficacy of a nano-metal organic framework by interior functionalization with 3-Hydroxy-2-pyridinone. Chin. J. Chem..

[20] Xu W, Kang Y, Jiao L, Wu Y, Yan H (2020). Tuning atomically dispersed Fe sites in metal-organic frameworks boosts peroxidase-like activity for sensitive biosensing. Nano-Micro Lett..

[21] Wu J, Wang Z, Jin X, Zhang S, Li T, Zhang Y (2021). Hammett relationship in oxidase-mimicking metal-organic frameworks revealed through a protein-engineering-inspired strategy. Adv. Mater..

[22] Lu W, Wei Z, Gu Z-Y, Liu T-F, Park J (2014). Tuning the structure and function of metal–organic frameworks via linker design. Chem. Soc. Rev..

[23] Xue Z, Liu K, Liu Q, Li Y, Li M (2019). Missing-linker metal-organic frameworks for oxygen evolution reaction. Nat. Commun..

[24] Wang Y, Li J, Zhou Z, Zhou R, Sun Q (2021). Halo-fluorescein for photodynamic bacteria inactivation in extremely acidic conditions. Nat. Commun..

[25] Kresse G, Furthmüller J (1996). Efficiency of ab-initio total energy calculations for metals and semiconductors using a plane-wave basis set. Comput. Mater. Sci..

[26] Kresse G, Furthmüller J (1996). Efficient iterative schemes for ab initio total-energy calculations using a plane-wave basis set. Phys. Rev. B.

[27] Perdew JP, Burke K, Ernzerhof M (1996). Generalized gradient approximation made simple. Phys. Rev. Lett..

[28] Blöchl PE (1994). Projector augmented-wave method. Phys. Rev. B.

[29] Grimme S, Antony J, Ehrlich S, Krieg H (2010). A consistent and accurate ab initio parametrization of density functional dispersion correction (DFT-D) for the 94 elements H-Pu. J. Chem. Phys..

[30] Masciocchi N, Cairati P, Carlucci L, Mezza G, Ciani G (1996). 1996) Ab-initio X-ray powder diffraction structural characterization of co-ordination compounds: Polymeric [{MX2 (bipy)}n] complexes (M= Ni or Cu; X= Cl or Br; bipy= 4, 4’-bipyridyl. J. Chem. Soc Dalton Trans..

[31] Sheng Y, Abreu IA, Cabelli DE, Maroney MJ, Miller A-F (2014). Superoxide dismutases and superoxide reductases. Chem. Rev..

[32] Hammer B, Norskov JK (1995). Why gold is the noblest of all the metals. Nature.

[33] Nørskov JK, Abild-Pedersen F, Studt F, Bligaard T (2011). Density functional theory in surface chemistry and catalysis. Proc. Natl. Acad. Sci..

[34] Shen X, Liu W, Gao X, Lu Z, Wu X (2015). Mechanisms of oxidase and superoxide dismutation-like activities of gold, silver, platinum, and palladium, and their alloys: a general way to the activation of molecular oxygen. J. Am. Chem. Soc..

[35] Wang Z, Wu J, Zheng J-J, Shen X, Yan L (2021). Accelerated discovery of superoxide-dismutase nanozymes via high-throughput computational screening. Nat. Commun..

[36] Liu J, Zou S, Xiao L, Fan J (2014). Well-dispersed bimetallic nanoparticles confined in mesoporous metal oxides and their optimized catalytic activity for nitrobenzene hydrogenation. Catal. Sci. Technol..

[37] Zhang R, Xue B, Tao Y, Zhao H, Zhang Z (2022). Edge-site engineering of defective Fe-N_4_ nanozymes with boosted catalase-like performance for retinal vasculopathies. Adv. Mater..

[38] Ray PD, Huang B-W, Tsuji Y (2012). Reactive oxygen species (ROS) homeostasis and redox regulation in cellular signaling. Cell. Signal..

[39] Fuse Y, Kobayashi M (2017). Conservation of the Keap1-Nrf2 system: an evolutionary journey through stressful space and time. Molecules.

[40] Simon H-U, Haj-Yehia A, Levi-Schaffer F (2000). Role of reactive oxygen species (ROS) in apoptosis induction. Apoptosis.

[41] Shroff EH, Snyder C, Chandel NS (2007). Bcl-2 family members regulate anoxia-induced cell death. Antioxid. Redox Signal..

[42] Ola MS, Nawaz M, Ahsan H (2011). Mol. Cell. Biochem..

[43] Yue J, López JM (2020). Understanding MAPK signaling pathways in apoptosis. Int. J. Mol. Sci..

[44] Liu W, Schultz KM, Zhang K, Sasman A, Gao F (2014). In vivo corneal neovascularization imaging by optical-resolution photoacoustic microscopy. Photoacoustics.

[45] Dua HS, Ting DSJ, Al Saadi A, Said DG (2020). Chemical eye injury: Pathophysiology, assessment and management. Eye.

[46] Chen Y, Yang W, Zhang X, Yang S, Peng G (2016). MK2 inhibitor reduces alkali burn-induced inflammation in rat cornea. Sci. Rep..

[47] Gordon S, Hamann J, Lin H-H, Stacey M (2011). F4/80 and the related adhesion-GPCRs. Eur. J. Immunol..

[48] Tomasek JJ, McRae J, Owens GK, Haaksma CJ (2005). Regulation of α-smooth muscle actin expression in granulation tissue myofibroblasts is dependent on the intronic CArG element and the transforming growth factor-β1 control element. Am. J. Pathol..

[49] Zhu S, Gong L, Li Y, Xu H, Gu Z (2019). Safety assessment of nanomaterials to eyes: An important but neglected issue. Adv. Sci..

