# Supplementary material for: A Rational Design of Metal–Organic Framework Nanozyme with High-Performance Copper Active Centers for Alleviating Chemical Corneal Burns
Source: Nanomicro Lett. 2023 Apr 30;15:112. doi: 10.1007/s40820-023-01059-9 (PMC10149557; doi:10.1007/s40820-023-01059-9)
Supplement: Supplementary file 1 — Supplementary file1 (PDF 1465 KB) [file 40820_2023_1059_MOESM1_ESM.pdf]

Supporting Information for

A Rational Design of Metal-Organic Framework Nanozyme with High-Performance Copper Active Centers for Alleviating Chemical Corneal Burns

Yonghua Tang[1, #], Yi Han[2, #], Jiachen Zhao[1], Yufei Lv[4], Chaoyu Fan[1], Lan Zheng[2], Zhisen Zhang[1], Zuguo Liu[2, 4, *], Cheng Li[2, 4, *], and Youhui Lin[1, 3, *]

[1] Department of Physics, Research Institute for Biomimetics and Soft Matter, Fujian Provincial Key Laboratory for Soft Functional Materials Research, Xiamen University, Xiamen 361005, P. R. China

[2] Fujian Provincial Key Laboratory of Ophthalmology and Visual Science & Ocular Surface and Corneal Diseases, Eye Institute & Affiliated Xiamen Eye Center, School of Medicine, Xiamen University, Xiamen 361102, P. R. China

[3] National Institute for Data Science in Health and Medicine, Xiamen University, Xiamen 361102, P. R. China

[4] Postdoctoral mobile station of Basic Medical Sciences, Hengyang Medical School; Department of Ophthalmology, the First Affiliated Hospital of University of South China, University of South China, Hengyang, Hunan, 421001, P. R. China

[#]Yonghua Tang and Yi Han contributed equally to this work.

*Corresponding authors. E-mail: linyouhui@xmu.edu.cn (Youhui Lin), cheng-li@xmu.edu.cn (Cheng Li), zuguoliu@xmu.edu.cn (Zuguo Liu)

Supplementary Figures and Tables

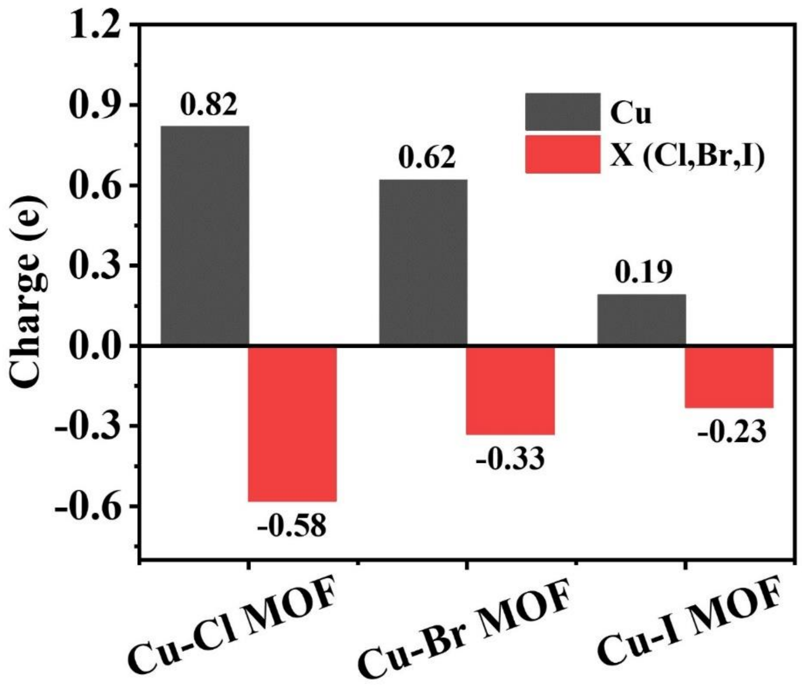

Fig. S1 Bader charge of Cu and X in Cu-X MOF

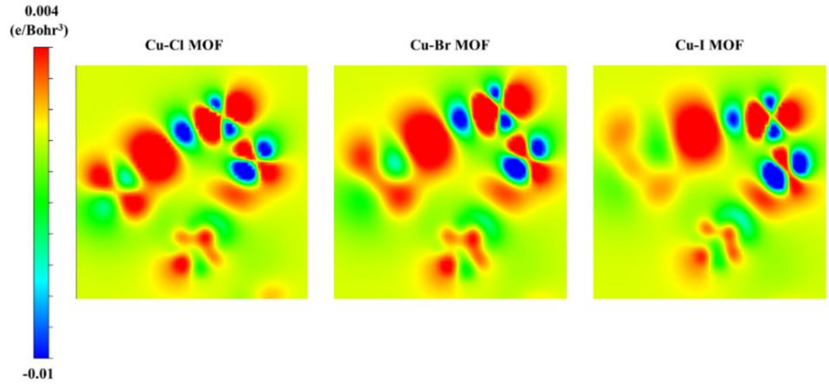

Fig. S2 The two-dimensional charge density difference diagram of Cu-X MOF

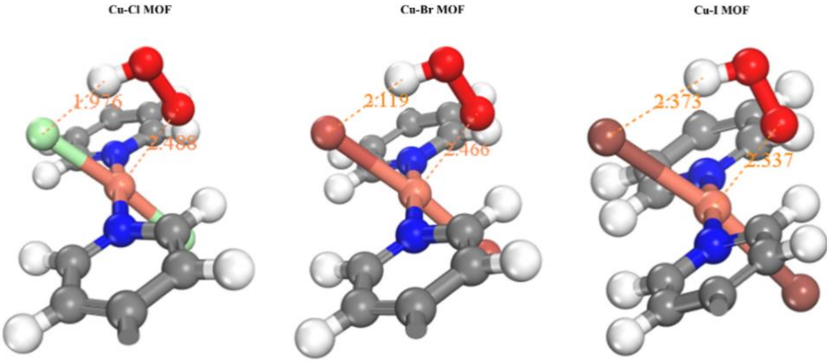

Fig. S3 The conformation of adsorbed •OOH on the Cu-X MOF model and the distances of Cu from O and X from H. (Unit: Å)

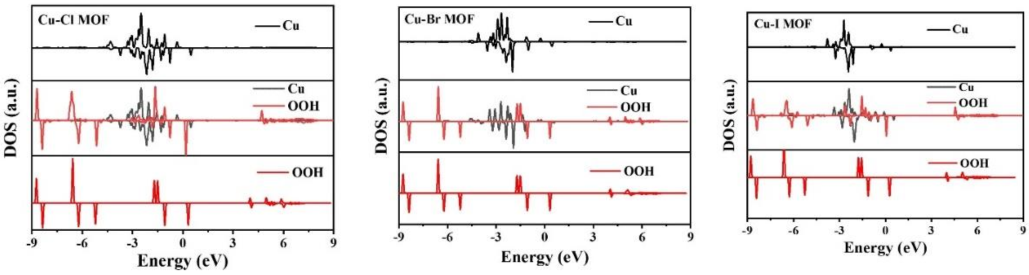

Fig. S4 Projection density of states of adsorbed •OOH on the Cu-X MOF model

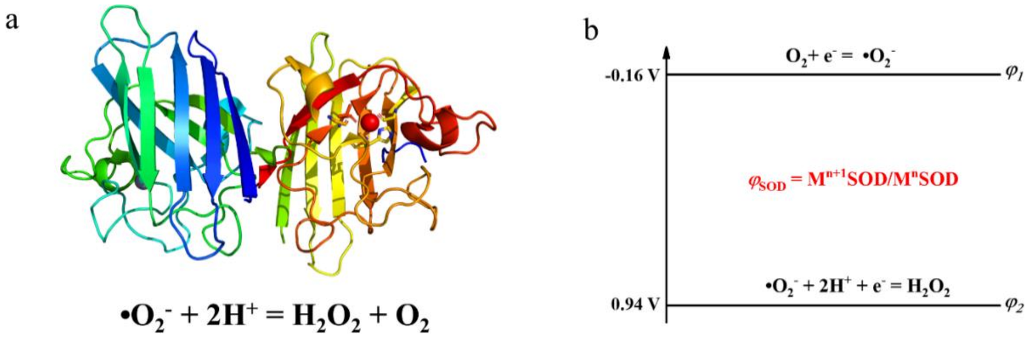

Fig. S5 a Superoxide dismutase (SOD) scavenges $\bullet O_2^-$ through a catalytic delocalization reaction. **b** A reduction potential model to predict the catalytic activity of SOD

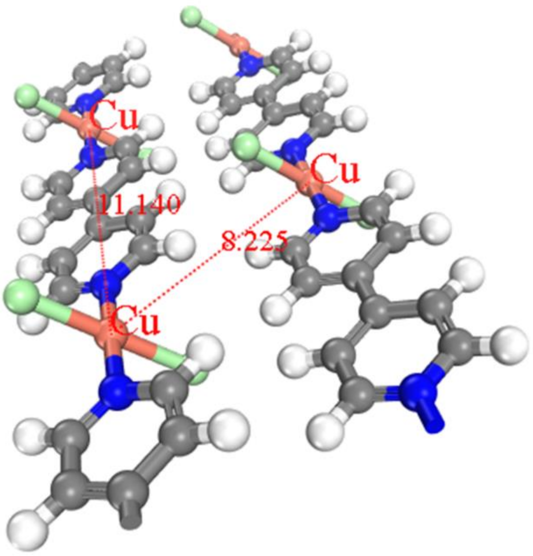

Fig. S6 Distance between the two nearest copper active sites. (Unit: Å)

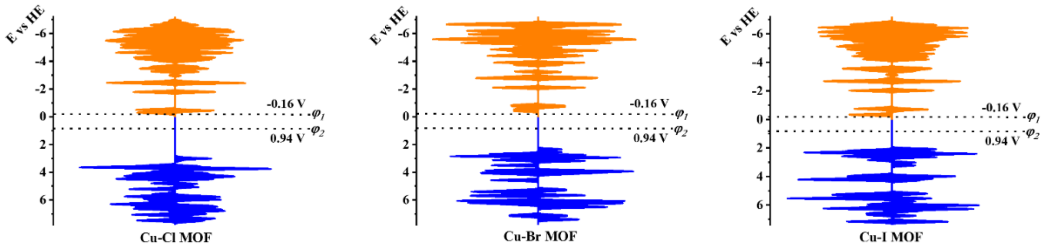

Fig. S7 Energy level of the Cu-X MOFs surface relative to the hydrogen electrode (HE) potential

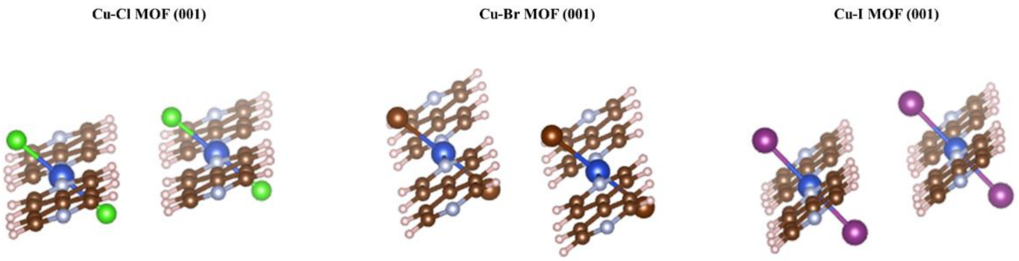

Fig. S8 Structures of Cu-X MOFs (001) surfaces

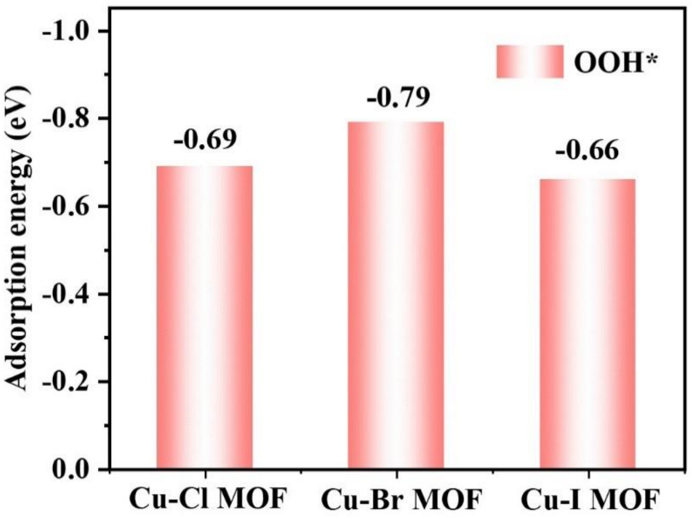

Fig. S9 Adsorption energy of •OOH on the surface of Cu-X MOFs

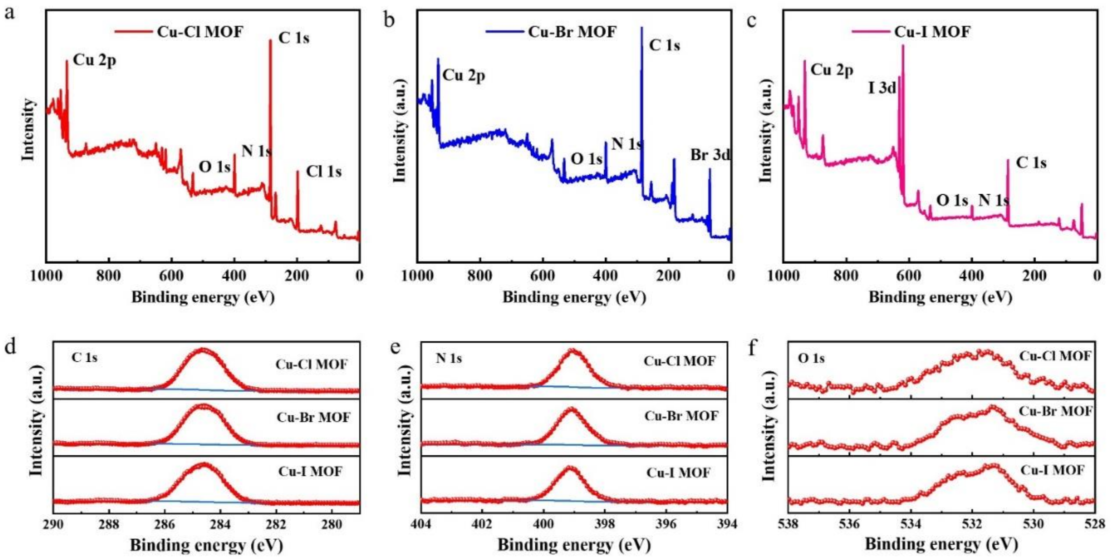

Fig. S10 a-c The XPS survey spectrum of different Cu-X MOFs. **d-e** The high-resolution C 1s, N 1s and O 1s XPS spectra of Cu-X MOFs

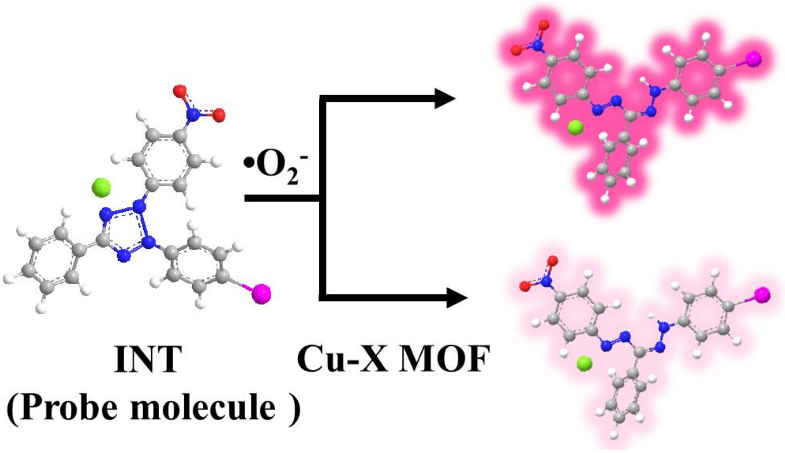

Fig. S11 Diagram of the superoxide radical (•O_2^-) capture mechanism by iodonitrotetrazolium chloride (INT)

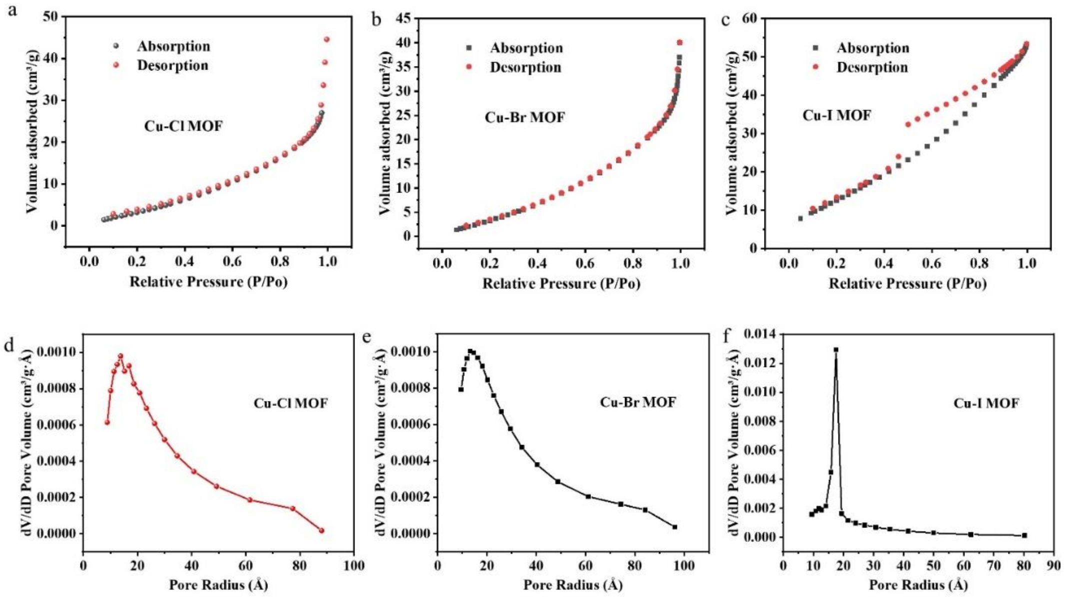

Fig. S12 N_2 adsorption-desorption isotherms **a-c** and pore size distribution **d-f** of Cu-X MOFs

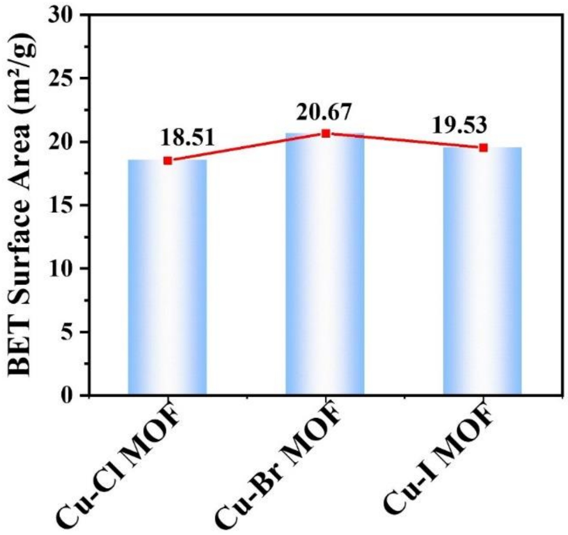

Fig. S13 Specific surface area data of Cu-X MOFs

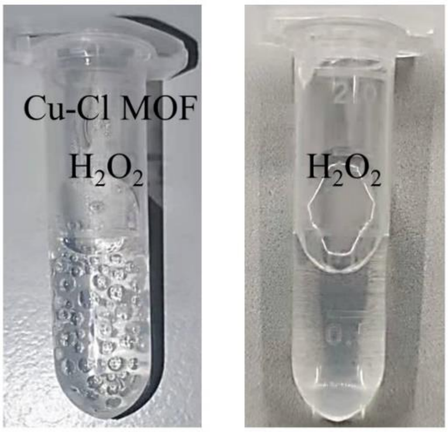

Fig. S14 Photograph of the catalytic formation of oxygen bubbles after incubation with and without Cu-Cl MOF (50 μg/mL) in a 100 mM H_2O_2 solution for 2 h

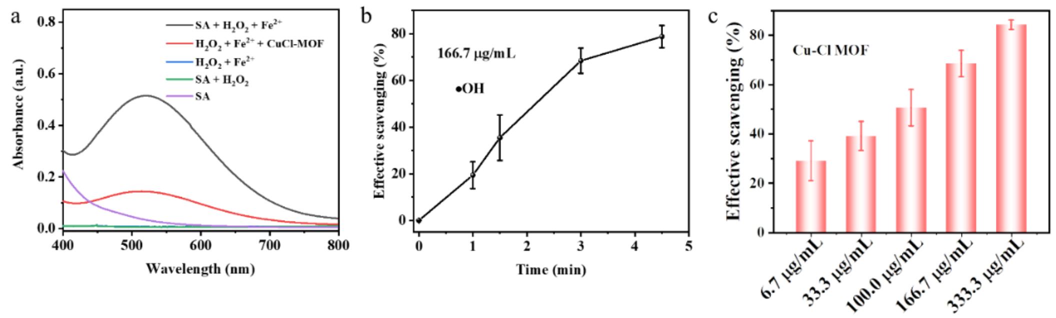

Fig. S15 **a** Absorption spectra of salicylic acid after reaction with Fe^{2+}/H_2O_2 in the absence and presence of Cu-Cl MOF nanozyme. **b** Hydroxyl radical removal efficiency curve with time. **c** The removal efficiency of •OH different masses of Cu-Cl MOF

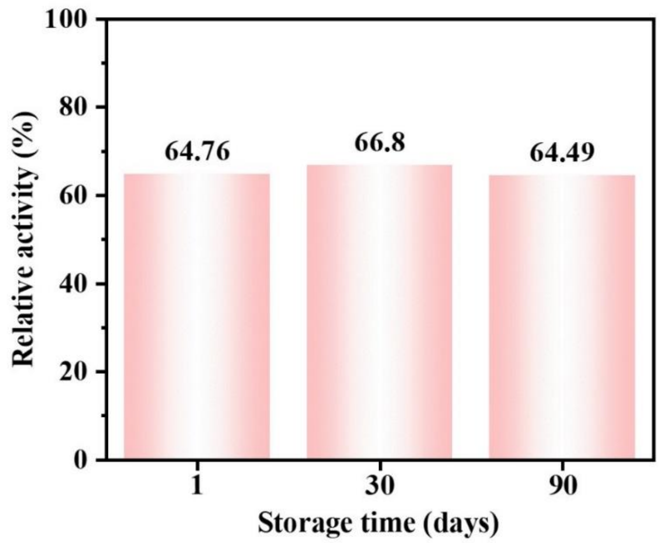

Fig. S16 SOD-like activity of Cu-Cl MOF at different storage times

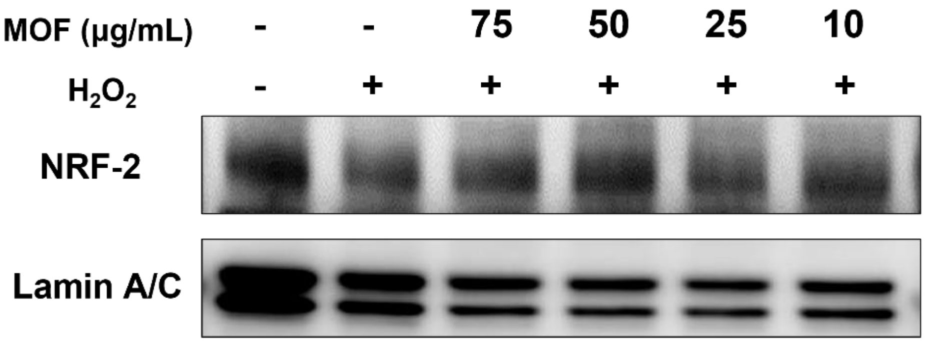

Fig. S17 Nuclear protein levels of NRF2 after incubation with varying concentrations of Cu-Cl MOF

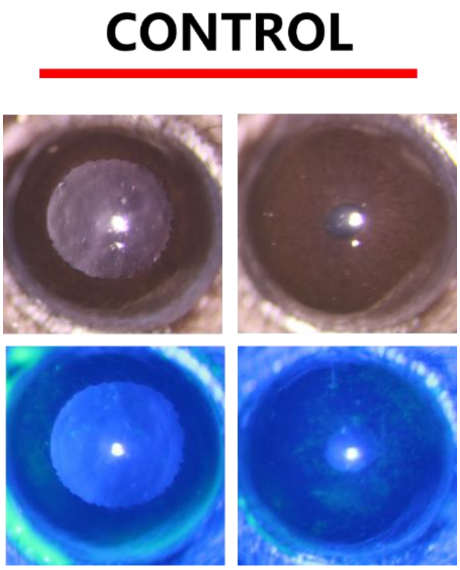

Fig. S18 Broad beam lighting and fluorescein staining images of the ocular surface of an alkali-burned control

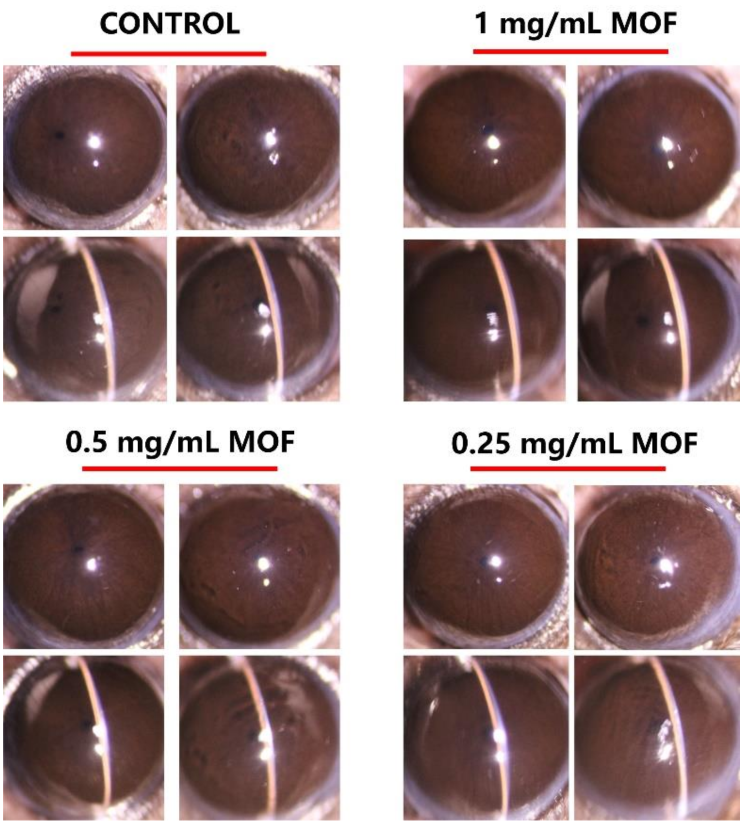

Fig. S19 Broad beam lighting and fluorescein staining images of the ocular surface after continuous administration of Cu-Cl MOF based eye drops in normal mice

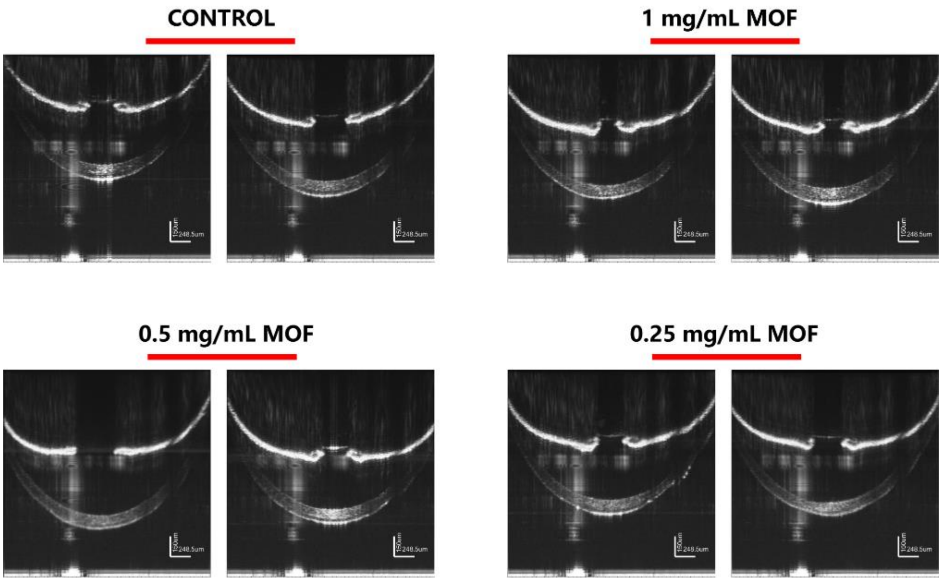

Fig. S20 AS-OCT images of the retina after continuous administration of Cu-Cl MOF based eye drops in normal mice

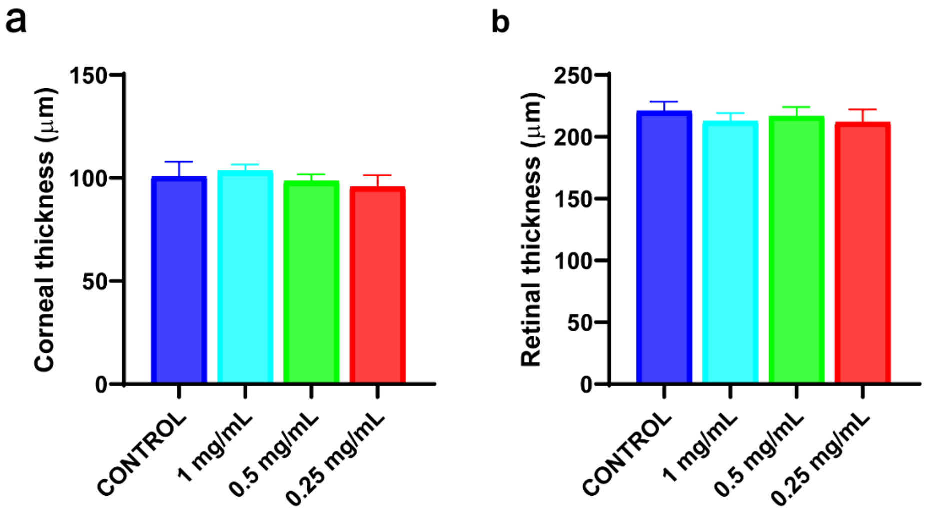

Fig. S21 Quantitative statistics of corneal thickness and retinal thickness measured and analyzed by OCT

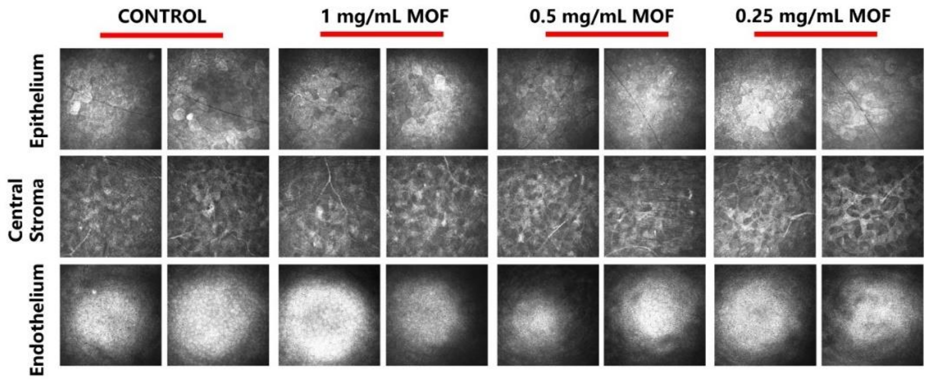

Fig. S22 *In vivo* confocal microscopy images of corneal epithelium, central corneal stroma, and corneal endothelium of ocular surface after continuous Cu-Cl MOF based eye drops in normal mice

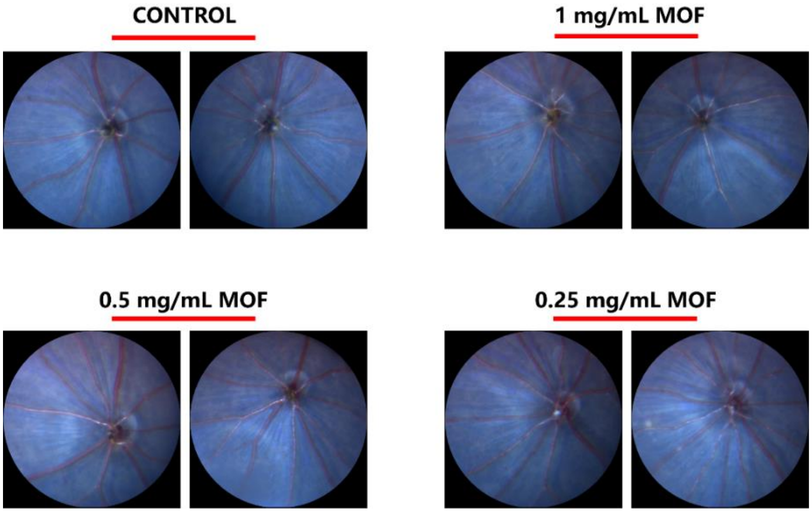

Fig. S23 After continuous administration of Cu-Cl MOF based eye drops in normal mice, fundus images of eyes were captured

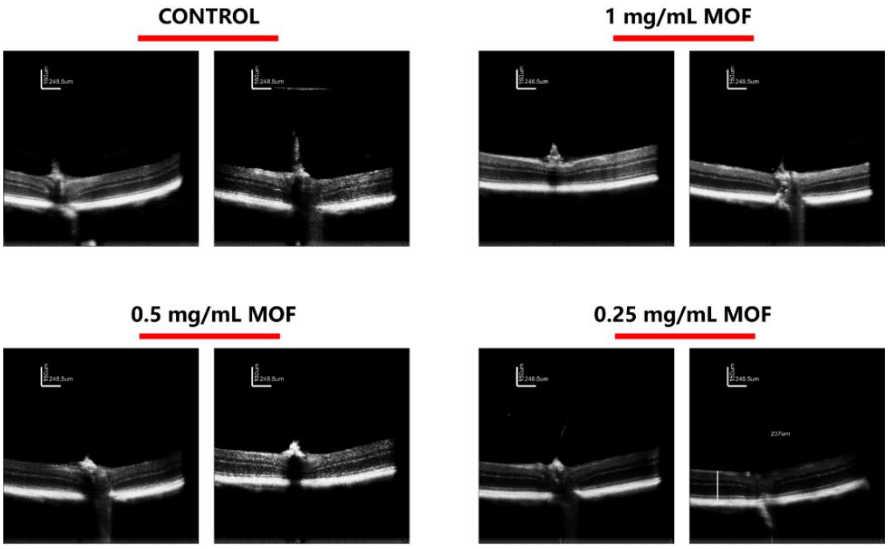

Fig. S24 PS-OCT images of the retina after continuous administration of Cu-Cl MOF based eye drops in normal mice

Table S1 Comparison of the specific activities of the Cu-Cl MOF and other Cu-based nanozymes

Sample	Specific activities (U/mg, SOD)
Cu-Cl MOF (This work)	-63.5
CuWK[1]	-4.1
CuSF[1]	-2.2
Commercial Cu_2O	-1.6
Commercial CuO	~0
Commercial Cu $(OH)_2$	~0

Specific activities =V/(Ɛ×l) ×(ΔA/Δt)/m, V is the total volume of the reaction solution (μL); ε is the molar absorption coefficient of the colorimetric substrate; l is the path length of light travelling in the cuvette (cm); A is the absorbance after subtraction of the maximum absorbance, and ΔA/Δt is the initial rate of change in absorbance; m is the nanozyme weight (mg) of each assay. The "-" represents the reduction of $•O_2^-$.

Supplementary References

[1] F. Xu, Y.H. Tang, H. Wang, H.B. Deng, Y.Y. Huang et al., Using wool keratin derived metallo-nanozymes as a robust antioxidant catalyst to scavenge reactive oxygen species generated by smoking. Small 18 2201205 (2022). https://doi.org/10.1002/smll.202201205
